# The effect of low-dose IL-2 and Treg adoptive cell therapy in patients with type 1 diabetes

**DOI:** 10.1172/jci.insight.147474

**Published:** 2021-09-22

**Authors:** Shen Dong, Kamir J. Hiam-Galvez, Cody T. Mowery, Kevan C. Herold, Stephen E. Gitelman, Jonathan H. Esensten, Weihong Liu, Angela P. Lares, Ashley S. Leinbach, Michael Lee, Vinh Nguyen, Stanley J. Tamaki, Whitney Tamaki, Courtney M. Tamaki, Morvarid Mehdizadeh, Amy L. Putnam, Matthew H. Spitzer, Chun Jimmie Ye, Qizhi Tang, Jeffrey A. Bluestone

**Affiliations:** 1 Sean N. Parker Autoimmune Research Laboratory and; 2 Diabetes Center, UCSF, San Francisco, California, USA.; 3 Parker Institute for Cancer Immunotherapy, San Francisco, California, USA.; 4 Department of Microbiology and Immunology,; 5 Biomedical Sciences Graduate Program,; 6 Department of Otolaryngology,; 7 Department of Microbiology and Immunology, and; 8 Helen Diller Family Comprehensive Cancer Center, UCSF, San Francisco, California, USA.; 9 Chan Zuckerberg Biohub, San Francisco, California, USA.; 10 Institute for Human Genetics and; 11 Medical Scientist Training Program, UCSF, San Francisco, California, USA.; 12 Department of Immunobiology and Internal Medicine, Yale University, New Haven, Connecticut, USA.; 13 Division of Pediatric Endocrinology, Department of Pediatrics;; 14 Department of Laboratory Medicine;; 15 Parnassus Flow Cytometry Core; and; 16 Transplantation Research Lab, Division of Transplant Surgery, Department of Surgery, UCSF, San Francisco, California, USA.

**Keywords:** Autoimmunity, Clinical Trials, Diabetes, Immunotherapy, T cells

## Abstract

**BACKGROUND:**

A previous phase I study showed that the infusion of autologous Tregs expanded ex vivo into patients with recent-onset type 1 diabetes (T1D) had an excellent safety profile. However, the majority of the infused Tregs were undetectable in the peripheral blood 3 months postinfusion (Treg-T1D trial). Therefore, we conducted a phase I study (TILT trial) combining polyclonal Tregs and low-dose IL-2, shown to enhance Treg survival and expansion, and assessed the impact over time on Treg populations and other immune cells.

**METHODS:**

Patients with T1D were treated with a single infusion of autologous polyclonal Tregs followed by one or two 5-day courses of recombinant human low-dose IL-2 (ld-IL-2). Flow cytometry, cytometry by time of flight, and 10x Genomics single-cell RNA-Seq were used to follow the distinct immune cell populations’ phenotypes over time.

**RESULTS:**

Multiparametric analysis revealed that the combination therapy led to an increase in the number of infused and endogenous Tregs but also resulted in a substantial increase from baseline in a subset of activated NK, mucosal associated invariant T, and clonal CD8^+^ T cell populations.

**CONCLUSION:**

These data support the hypothesis that ld-IL-2 expands exogenously administered Tregs but also can expand cytotoxic cells. These results have important implications for the use of a combination of ld-IL-2 and Tregs for the treatment of autoimmune diseases with preexisting active immunity.

**TRIAL REGISTRATION:**

ClinicalTrials.gov NCT01210664 (Treg-T1D trial), NCT02772679 (TILT trial).

**FUNDING:**

Sean N. Parker Autoimmune Research Laboratory Fund, National Center for Research Resources.

## Introduction

Type 1 diabetes mellitus (T1D) is an increasingly prevalent, yet still poorly understood, medical condition with devastating long-term complications, such as retinopathy, neuropathy, cardiovascular disease, and renal failure. The disease pathogenesis, which is influenced by genetic and environmental factors, is attributed to the loss of immune tolerance, which results in the development and inadequate control of pathogenic, autoreactive T cells that recognize and destroy islet β cells (1–3). Tregs play a predominant role in maintaining peripheral tolerance, and defects in their function have been described in patients with T1D (4–6). For instance, several studies have shown that the Tregs in the peripheral blood of patients with T1D can produce effector cytokines, such as IFN-γ (7, 8), and fail to regulate activated T effector cells (9, 10).

Adoptive transfer of Tregs has been shown to reverse T1D in a mouse model of spontaneous disease (11). Based on these results, we previously conducted a phase I trial (NCT01210664 — Treg-T1D) testing expanded autologous polyclonal Tregs as therapeutics aimed to restore tolerance in patients with recent-onset T1D (12). We demonstrated that Tregs could be efficiently isolated from peripheral blood of patients with T1D and expanded 300- to 2000-fold within a 2-week period. Moreover, the dysfunction that was associated with Tregs from patients with T1D was improved during culture. The expanded Tregs expressed higher levels of cytotoxic T lymphocyte–associated protein 4 (CTLA-4) and latency-associated peptide, and a defect in IL-2–induced STAT5 signaling was reversed. Infusion of the Tregs in patients with recent-onset T1D had an excellent safety profile, and about 50% of recipients maintained their insulin production for 2 years. Moreover, we showed that the adoptively transferred Treg cells remained phenotypically stable in the blood for at least 1 year postinfusion. However, there was a considerable and rapid decline in the percentage of infused Tregs in the peripheral blood, with, on average, over 75% of the infused Tregs absent from the circulation within 90 days. We hypothesized that the rapid decline might be attributed, at least in part, to a lack of IL-2 in patients with T1D, especially because GWAS have suggested a genetic link to IL-2 deficiency in this autoimmune setting (13).

IL-2 is a survival and growth factor cytokine essential for Treg development and function, inducing effective STAT5-mediated signaling to achieve full functionality and survival (14–17). In fact, genetic deficiency in the IL-2/IL-2R pathway leads to systemic autoimmunity (18). Tregs express the highest level of high-affinity α chain (CD25) as part of the trimeric receptor (α, β, γ) receptor complex, which make them highly responsive to even small amounts of the cytokine in the biological environment. In vivo administration of low doses of IL-2 can expand Tregs and ameliorate disease in multiple autoimmune disease models in mice. For instance, low-dose IL-2 (ld-IL-2) prevents and reverses diabetes in the spontaneous NOD mouse model (19, 20). Early clinical studies suggested that ld-IL-2 blocks the progression of graft-versus-host disease, systemic lupus erythematosus, and autoimmune hepatitis (21–24). However, IL-2 is also a growth factor for conventional T, NK, and other potentially pathogenic cells due to the ubiquitous expression of the IL-2 receptor subunits in multiple activated immune cell populations. Hence, the dual activity of IL-2 put the drug at the crossroads of tolerance versus activation, and predicting its therapeutic effects remains uncertain (25).

Thus, we designed a phase I study to determine if a combination of autologous Treg and ld-IL-2 therapies could demonstrate significant biologic activity based on multiparametric biomarker analyses of peripheral blood samples collected longitudinally from patients with T1D enrolled in the study.

## Results

### Study design, patient population, and analysis of metabolic function levels.

The phase I trial was designed as an open-label, dose-escalating study conducted at 2 sites, Yale University and the UCSF. Nine patients met the eligibility criteria. Seven were enrolled in the first cohort and 2 in the second cohort (Supplemental Figure 1; supplemental material available online with this article; https://doi.org/10.1172/jci.insight.147474DS1). A schematic representation of the Treg dose escalation plan and the approved plan for Proleukin (IL-2) dosing are illustrated in Supplemental Figure 2, A and B, respectively. Representation of an individual’s planned participation is shown in Supplemental Figure 2C. All the participants in the first cohort received the prescribed infusion of the expanded Tregs (3 × 10^6^/kg) (Supplemental Table 1). There were 2 dosing cohorts planned, each consisting of 6–8 subjects treated with a single infusion of polyclonal Tregs and ld-IL-2 at the doses shown in Supplemental Table 1. Supplemental Table 1 and Supplemental Data File 3 show the demographics and baseline hemoglobin A1c (HbA1c) levels. The mean age was 25.7 ± 4.7 years, and the mean disease duration was 46.1 ± 15.7 weeks at the time of screening. Metabolic assessment of all patients from cohort 1 and cohort 2 was followed up to 104 weeks and 78 weeks, respectively.

**Figure 1:**
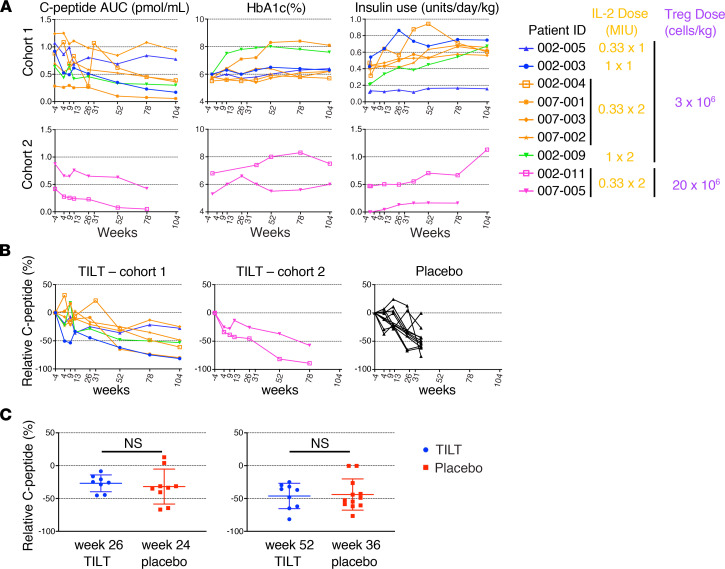
Metabolic assessments. (**A**) (Left column) C-peptide AUC is reported for fasting 4-hour mixed meal tolerance test (MMTT) without carbohydrate restriction for 3 days preceding testing. The target glucose level at the start of the test was between 70 and 200 mg/dL. Regular insulin or short-acting insulin analogs were allowed up to 6 and 2 hours before the test, respectively, to achieve the desired glucose level. The baseline blood samples (−10 minutes and 0 minutes) were drawn, and then patients drank Boost high protein nutritional energy drink (Nestle Nutrition) at 6 kcal/kg (1 kcal/mL) to a maximum of 360 mL. Blood was drawn at 15, 30, 60, 90, 120, 150, 180, 210, and 240 minutes following Boost dose. C-peptide AUC was calculated using the trapezoid rule. (Middle column) Hemoglobin A1c (HbA1c). (Right column) Insulin use. Insulin use for the 3 days immediately preceding the scheduled visit was self-reported. The average total insulin (long acting + short acting) use per day normalized to weight is reported. Table shows Treg and IL-2 dosage of each patient. MIU, million international units. (**B**) Percentage of relative C-peptide loss up to 104 and 78 weeks in patients from cohorts 1 and 2, respectively, of the TILT trial (2 left graphs) and from the placebo cohort of the AIDA and NT-14 trial (right graph). (**C**) Comparison of percentage of relative C-peptide loss at the indicated time point between the patients from TILT and placebo groups.

Changes in β cell function over time were assessed by measuring the mixed meal tolerance test–stimulated (MMTT-stimulated) 4-hour C-peptide AUC, HbA1c, and insulin usage. The adverse events were limited to local events at the site of IL-2 injection (Supplemental Data File 1). The stimulated C-peptide levels decreased in all the patients within the first 30 days of treatment (Figure 1A). After 30 days, the stimulated C-peptide levels stabilized or increased slightly in 2 participants but continued to decline in the other 7 participants. Consistent with this, the insulin usage and HbA1c increased in 6/7 and 5/7 participants, respectively (Figure 1A). Retrospective analyses, made available after the initiation of the TILT study, showed a similar C-peptide decay in the TILT patients when compared to the C-peptide responses in age-matched individuals who received placebo drugs in the anti-interleukin 1 inhibitor (Anakinra) AIDA and the anti-IL-1β Canakinumab TrialNet TN-14 trials (26, 27). Thus, the TILT combination therapy did not improve islet metabolic function in any of the first 9 patients (Figure 1, B and C). These results suggested that the number of participants planned for the trial would not be sufficient to demonstrate efficacy, which led to termination of the trial after the second patient in cohort 2 was treated.

### Longitudinal tracking of Treg postinfusion.

**Figure 2:**
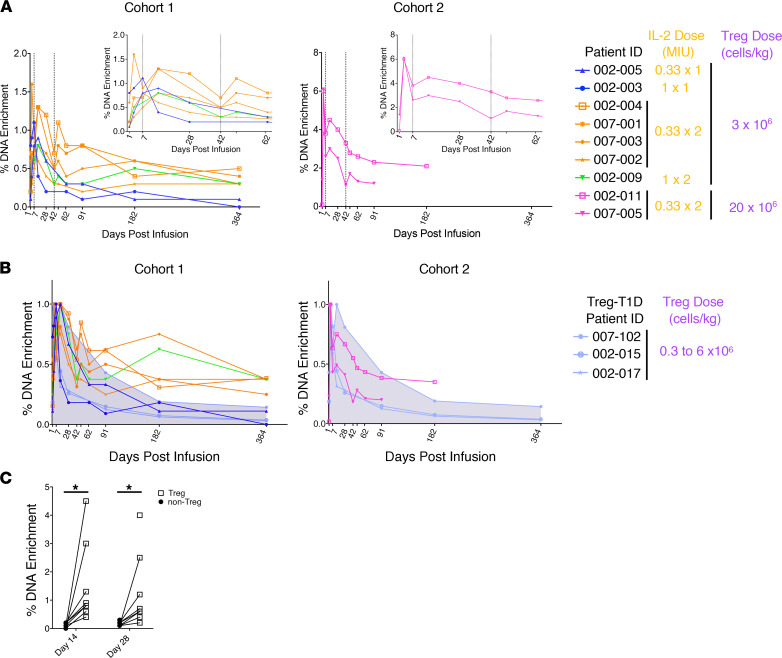
Longitudinal tracking of in vitro–expanded Tregs postinfusion. (**A**) Graphs show the percentage of DNA enrichment with deuterium (^2^H) in PBMC sorted Treg cells from TILT trial patients. Enlarged view of the ^2^H labeling kinetics up to 63 days is represented in the upper right of each graph. Black dashed lines indicate the fifth day of each IL-2 infusion course. Table shows Treg and IL-2 dosage of each patient. (**B**) Graphs show the percentage of deuterated DNA enrichment normalized to the maximum value in total PBMCs over time in each patient from the TILT trial. Light blue lines and gray areas show superimposition to normalized percentage of deuterated DNA enrichment of the T1D trial. Table shows Treg dosage. (**C**) Percentage of ^2^H level in postinfusion sorted non-Tregs versus Tregs in TILT trial patients. Paired 2-tailed *t* tests were performed in order to assess statistical significance. *P < 0.05.

In our previous studies, in vitro–expanded Tregs retained a stable functional phenotype after infusion but declined precipitously as a percentage of total T cells in the peripheral blood after 1 month. We had hypothesized that the ld-IL-2 treatment might increase the functional phenotype and persistence of the adoptively transferred Treg population in vivo. To track the effect of ld-IL-2 on the infused Tregs’ persistence, Treg cell DNA was labeled with deuterium (^2^H) during the ex vivo expansion phase, and percentage of ^2^H-enriched DNA in PBMC sorted Tregs was measured by mass spectrometry. Figure 2A shows detailed kinetics of ^2^H-enriched DNA of Tregs isolated from each patient at the indicated time point. In most patients, there was an increase in the percentage of ^2^H-labeled Tregs after each 5-day course of IL-2 (at day 7 and day 43, shown with dashed lines) (Figure 2A, small graphs). The increased percentage of ^2^H-labeled Tregs was transient and declined by 91 days. Depending on the participant, this occurred ~6–12 weeks after the last course of IL-2 treatment. Figure 2B shows the level of ^2^H enrichment normalized to the maximum value of each patient. Since the ^2^H labeling would be expected to dilute if the cells went into cycle, it seemed likely that the increase in the percentage of ^2^H label after IL-2 treatment reflected changes in Treg trafficking or survival rather than proliferation. A comparison of the kinetics of ^2^H enrichment in the peripheral blood Tregs of TILT trial participants with analyses performed in the previous Treg-T1D trial in participants treated with a similar number of infused Tregs (as described in Supplemental Table 1) showed that there was a longer term maintenance of the cells in the peripheral blood up to a year in the 6 patients who received both doses of IL-2 (Figure 2B), supporting the hypothesis that IL-2 therapy enhanced long-term Treg survival.

To determine the stability of the adoptively transferred Tregs, at various time points, PBMCs were sorted into Treg (CD4^+^CD25^+^CD127^lo/–^) and non-Treg populations (CD4^+^CD25^lo^CD127^hi^ CD45RO^+^, CD45RO^+^CD62L^lo^, CD45RO^+^CD62L^hi^) and analyzed for the presence of ^2^H label. As seen in Figure 2C, all the ^2^H labeling remained associated with the CD4^+^CD25^+^CD127^lo/–^ Treg population over the first 28 days. Only background levels of ^2^H label were observed in non-Treg subsets (including activated T effector/memory cells), suggesting that the adoptive cell transferred (ACT) Tregs retained a stable phenotype over time (Figure 2C).

### Phenotypic changes of infused and endogenous Treg population after ld-IL-2 treatment.

**Figure 3:**
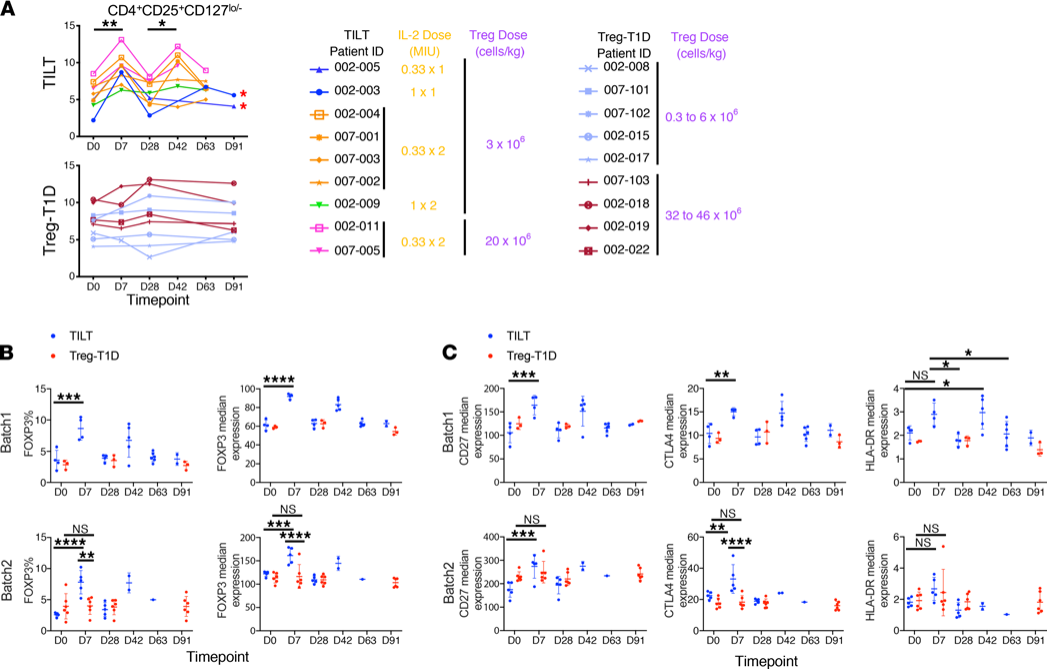
Low-dose IL-2 induces activation phenotype in the Treg subset at a protein level. (**A**) Graphs represent the percentage of Tregs (left column) by flow cytometry at the indicated time points. TILT trial patient data are shown in upper graphs, and the Treg-T1D trial patients are represented in the lower graphs. Red asterisks indicate patients who received only 1 dose of IL-2. Tables indicate dosage of IL-2 and Tregs for each patient. Paired 2-tailed *t* tests were performed in order to assess statistical significance. (**B** and **C**) Percentage of FOXP3^+^ and median expression of FOXP3^+^ as well as median expression of CD27, CTLA-4, and HLA-DR was assessed by CyTOF. Data were normalized; cell populations were gated manually in CellEngine. Populations were then exported for analysis in R, and marker expression values were then arsinh-transformed with a cofactor of 5 and represented in dot plots. The results are plotted into 2 separated batches (batches 1 and 2) due to batch effect affecting the comparison of the samples within the same analysis pipeline (batches layout of the samples, Supplemental Table 3). Asterisks indicate significance relative to the control group determined by 1-way ANOVA. ***P < 0.001; ****P < 0.0001.

The effects of ld-IL-2 on the Treg populations (both adoptively transferred and endogenous) in the 9 patients of the TILT trial were compared with those of 9 Treg-T1D study that had received a comparable or higher Treg dosage in the original phase I trial (NCT01210664-Treg-T1D trial) (Figure 3A legend table and Supplemental Table 1). A total of 76 frozen PBMC samples from the 18 patients, harvested at different time points (Supplemental Table 2), were analyzed by flow cytometry, cytometry by time of flight (CyTOF), single-cell RNA sequencing (RNA-Seq), and TCR sequencing (TCR-Seq).

Flow cytometric analyses of the whole PBMCs showed a significant increase in the percentage of the CD4^+^CD25^+^CD127^lo/–^ population in all the TILT patients after each ld-IL-2 treatment (Figure 3A). Similarly, CyTOF analysis showed an increase in the percentage and median expression of FOXP3 and also an increase in the median expression of activation markers such as CD27, CTLA-4, and HLA-DR in the Treg population after each course of ld-IL-2 treatment (day 7 and day 42) (Figure 3, B and C). The ld-IL-2–dependent increase of FOXP3^+^ Treg population was consistent with data shown in previous ld-IL-2 trials (28–30).

**Figure 4:**
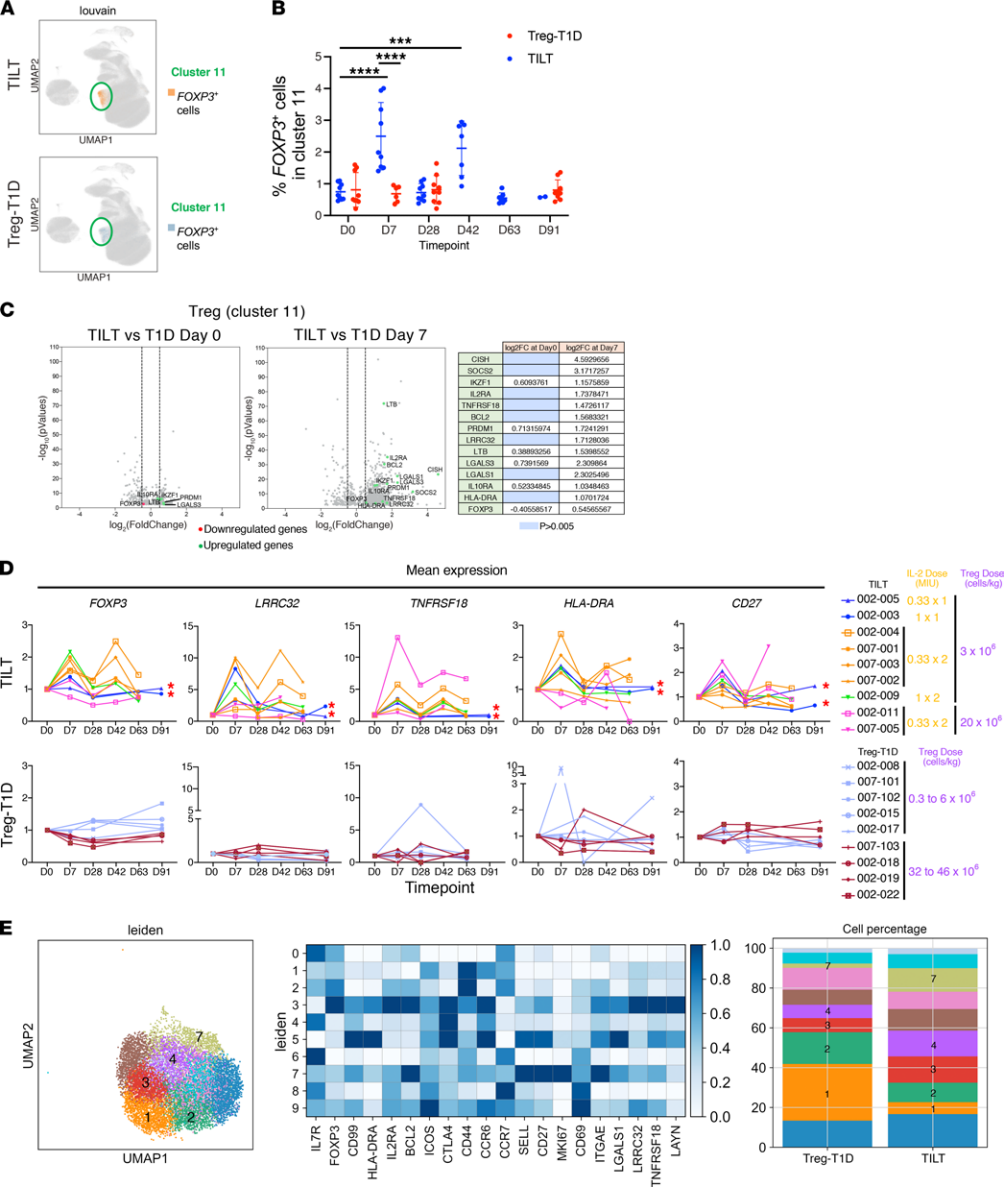
Low-dose IL-2 induces activation phenotype in the Treg subset at the mRNA level. 10x Genomics single-cell RNA-Seq data were analyzed by Scanpy package. (**A**) UMAPs show expression of FOXP3 in cluster 11 from UMAP in Supplemental Figure 2 in the TILT patient samples and Treg-T1D patient samples. (**B**) Volcano plots represent differential gene expression analysis of the Treg cell compartment (Supplemental Figure 2, cluster 11) from TILT and Treg-T1D patients at day 0 (left volcano plot) and day 7 (right volcano plot). Downregulated (red dots) and upregulated genes (green dots) are indicated in log_2_(fold change) (log2FC) with a *P* < 0.005. Gene expressions with *P* values greater than 0.005 were filtered out. Vertical dashed lines represent thresholds of log2FC of –0.6 and 0.6 corresponding to a fold change of 1.5 times. Table indicates the log2FC values of the indicated genes. Blue cells indicate nonsignificant genes filtered out due to a *P* > 0.005. (**C**) Dot plot shows longitudinal changes over time of percentage of FOXP3^+^ cells in cluster 11 for the 2 trials. Asterisks indicate significance relative to the control group determined by 1-way ANOVA. *P < 0.05; **P < 0.01; ***P < 0.001; ****P < 0.0001. (**D**) Graphs represent mean mRNA expression of the indicated genes normalized to day 0 for the patients of each clinical trial group (TILT in upper graphs and Treg-T1D in lower graphs). Red asterisks indicate patients that received only 1 dose of IL-2. (**E**) UMAP and leiden clustering of the Treg cluster 11. Heatmap shows Treg markers’ and activation markers’ mean expression in the indicated clusters. Stacked bar chart shows the percentage of cells in each cluster in Treg-T1D versus TILT patients.

To delve more deeply into phenotypic changes in immune subsets, CD45^+^ cells were isolated by FACS, combined into 30 pools, and sequenced in 3 distinct batches (Supplemental Table 4). The data from individual time points of each patient’s samples were then deconvoluted using the Demuxlet computational package (Supplemental Figure 3A) (31). The gene expression profile of more than 400,000 cells was integrated into a single uniform manifold approximation and projection (UMAP) plot clustering. The main immune cell populations were identified by known markers (Supplemental Figure 3B, including Tregs, B cells, NK cells, CD4 cells, CD8 cells, and dendritic cells/macrophages) as well as populations of granzyme B–activated (GZMB) and perforin-activated (PRF1) cells were illustrated using violin plots and UMAP plots (Supplemental Figure 3, C and D). UMAP analysis showed that FOXP3 expression was localized to cluster 11 (Supplemental Figure 3) in both the TILT and Treg-T1D samples (Figure 4A). Longitudinal analysis showed that each ld-IL-2 treatment (day 7 and day 42) resulted in an increased percentage of FOXP3^+^ T cells for all the patients from the TILT trial compared with Treg-T1D patients (Figure 4B). Expressed genes in the Treg cluster were compared between Treg-T1D and TILT samples. There were only a limited number of differences in gene expression between Treg-T1D and TILT Treg population before ld-IL-2 treatment (day 0) (Figure 4C, left volcano plot). In contrast, Tregs from TILT patients showed multiple upregulated mRNAs at day 7, after the first course of IL-2. The upregulated gene expression profiles included both phenotypic and functional Treg markers, including *FOXP3*, *TNFRSF18* (GITR), *LRRC32* (GARP), and *IKZF1* (IKAROS); activation marker genes, including *HLA-DRA* and *IL2RA*; and IL-2 signaling genes, including *CISH*, *SOCS2*, *BCL2*, and *PRDM1* (Figure 4C, right volcano plot). Moreover, corroborating CyTOF data (Figure 3, B and C), *FOXP3*, *LRRC32*, *TNFRSF18*, *HLA-DR*, and *CD27* mean mRNA expression was increased in Treg populations in the majority of the participants after ld-IL-2 treatment at day 7 and day 42 (Figure 4D; upper graphs) when compared with samples from patients in the Treg-T1D trial (Figure 4D, lower graphs).

Next, the cluster 11 was reanalyzed based on leiden clustering (UMAP, Figure 4E) to determine the heterogeneity of the Treg populations. A gene expression heatmap of the Treg population subclusters for all time points revealed increased CD44 and CCR7 expression in subclusters 1 and 2, matching the phenotype of an activated Treg population similar to activated Tregs seen in secondary lymphoid structures (32). Subcluster 3 cells represented the gene expression profile of memory Treg population with high expression of *CD25* (IL-2RA), *BCL2*, *CTLA4*, *ICOS*, and *CD27* and low expression of CD62L (*SELL*) and Ki-67 (*MKI67*) while subcluster 4 cells expressed low levels of *FOXP3*, but high levels of *ICOS* and *CTLA4*, consistent with a population of conventional T cells that upregulated *FOXP3*. Subcluster 7 Tregs expressed markers similarly to subcluster 3 but high levels of the proliferation marker, *MKI67* (Figure 4E, heatmap). Next, patient samples were examined for changes in Treg subclusters after Treg transfer and ld-IL-2 therapy. TILT patients showed a reduced number of activated Treg populations (subclusters 1 and 2) and an expanded percentage of memory and proliferating Treg cells (subclusters 3 and 7) (Figure 4E, stack bars chart). Interestingly, there was also an increase in subcluster 4, representing activated FOXP3^lo^ conventional T cells, suggesting that ld-IL-2 treatment resulted in increased percentages of Tregs, and some conventional T cells, expressing activated/memory markers. However, TCR analysis of the TCR repertoire and Gini coefficient calculation suggested that there was no selective clonal expansion of the Treg TCR repertoire (see below in Gini index graph of the CD8 population and Supplemental Table 5), consistent with the ld-IL-2 treatment broadly inducing the proliferation of activated Tregs and/or mobilized activated Tregs from the tissue into the circulation.

### Changes in innate cell subsets after Treg and ld-IL-2 treatment.

**Figure 5:**
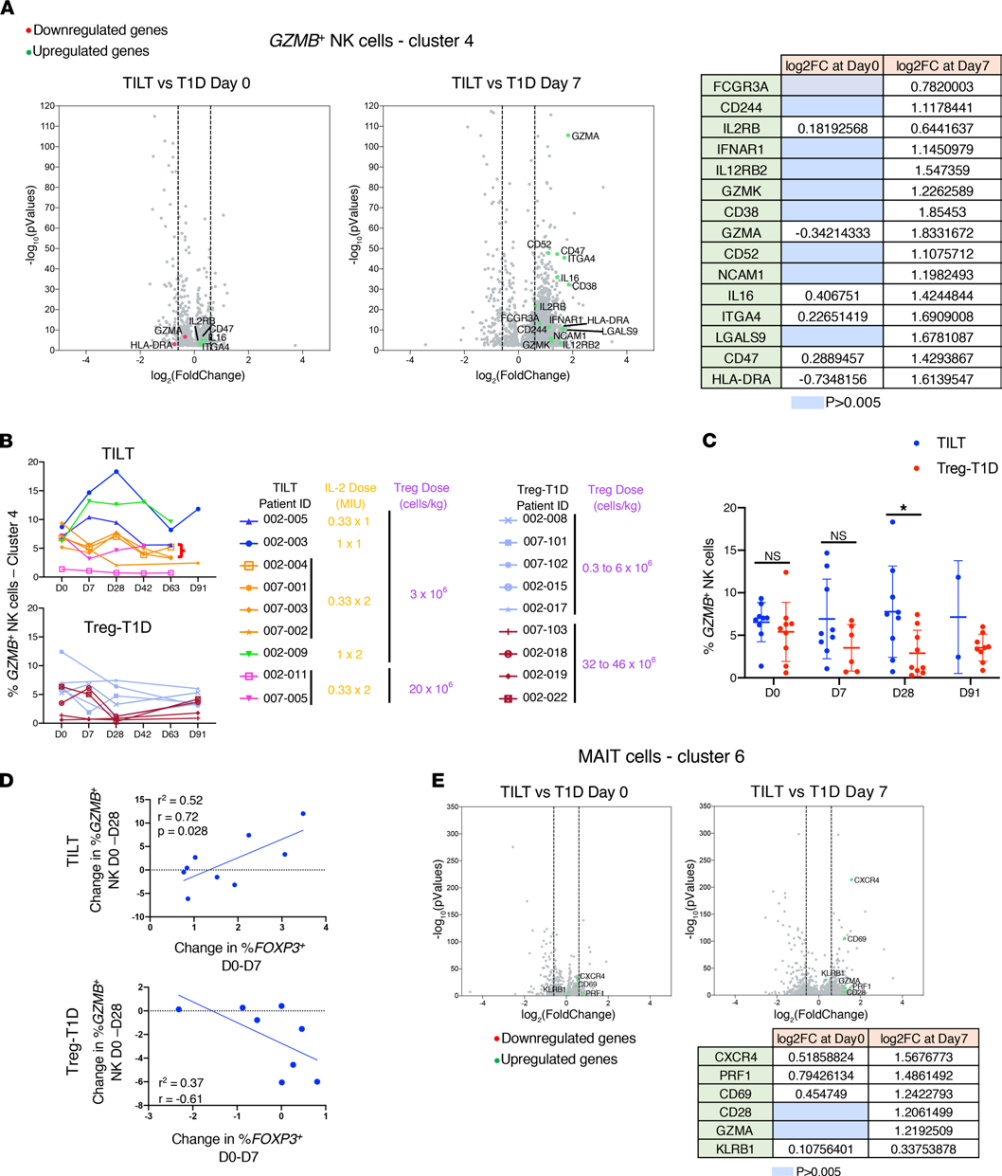
Low-dose IL-2 induces cytotoxic phenotype in the NK cell subset and mucosal invariant associated T cell subset. 10x Genomics single-cell RNA-Seq data were analyzed by Scanpy package. (**A**) Volcano plots represent differential gene expression analysis of the NK cell compartment (Supplemental Figure 2, cluster 4) from TILT and Treg-T1D patients at day 0 (left volcano plot) and day 7 (right volcano plot). Downregulated (red dots) and upregulated genes (green dots) are indicated in log2FC with a *P* < 0.005. Gene expressions with *P* values greater than 0.005 were filtered out. Vertical dashed lines represent thresholds of log2FC of –0.6 and 0.6 corresponding to a fold change of 1.5 times. Table indicates the log2FC values of the indicated genes. Blue cells indicate nonsignificant genes filtered out due to a *P* > 0.005. (**B**) Percentage of *GZMB*^+^ cells in the NK cluster (Supplemental Figure 2, cluster 4) were calculated and shown on upper graphs for TILT trial patients and lower graphs for Treg-T1D trial patients. Tables indicate dosage of IL-2 and Tregs for each patient. (**C**) Dot plot represents percentage over time of *GZMB*^+^ cells in NK clusters in TILT and Treg-T1D trial patients. Asterisks indicate significance relative to the control group determined by 1-way ANOVA. *P < 0.05. (**D**) Graphs show correlation of day 0 to day 28 changes in the percentage of *GZMB*^+^ NK and day 0 to day 7 changes in the percentage of *FOXP3*^+^ Treg cells in TILT patients (upper graph) and Treg-T1D patients (lower graph). (**E**) Volcano plots represent differential gene expression analysis of the MAIT cell compartment (Supplemental Figure 2, cluster 6) from TILT and Treg-T1D patients at day 0 (left volcano plot) and day 7 (right volcano plot). Table indicates the log2FC values of the indicated genes. Blue cells indicate nonsignificant genes filtered out due to a *P* > 0.005.

Multiparameter longitudinal single-cell analysis allowed the identification of other immune populations affected by ld-IL-2 and Treg treatment. Previous clinical trials have shown that ld-IL-2 treatment altered the percentage of CD56^+^ NK populations in the circulation (23, 29, 33–35). In this study, single-cell analysis of differentially expressed genes in the GZMB^+^ NK cluster (cluster 4 in Supplemental Figure 3) between Treg-T1D and TILT samples showed limited differences at day 0 while a large set of phenotypic and activation markers of mature NK cells was altered in peripheral blood cells isolated from TILT patients at day 7 after the first course of IL-2 (Figure 5A). Longitudinal analysis showed that most of the patients in both trials presented a similar percentage of GZMB^+^ NK cells at day 0. This population was increased in 3 TILT trial participants, while 2 patients showed no change and 4 patients showed a decline in this population after each ld-IL-2 course (Figure 5B, upper graph). In contrast, all the percentages of GZMB^+^ NK cells remained constant or decreased in samples from patients of the Treg-T1D trial (Figure 5B, lower graph). More importantly, at day 28, the percentage of GZMB^+^ NK cells significantly increased in all the TILT trial patients as compared with the patients in the Treg-T1D trial (Figure 5C). Interestingly, over time, the percentage of GZMB^+^ NK cells returned to baseline levels in all IL-2–treated patients. These data suggest that the administration of ld-IL-2 to patients with T1D led to changes in the phenotype and distribution of GZMB^+^ NK cells over time, likely a consequence of changes in expansion and trafficking in these patients. In fact, there was a positive correlation between changes in the Treg percentage and changes of the GZMB^+^ NK cells’ percentage after ld-IL-2 treatment (Figure 5D, upper graph). In contrast, those changes were not correlated in patients treated with Tregs only (Figure 5D, lower graph), suggesting that the ld-IL-2 treatment affected those 2 populations at the same time.

Based on the above observations, we examined whether other innate cell subsets were altered by the Treg and ld-IL-2 treatment. Interestingly, TCR analysis of the cluster 6 cells showed that the TRAV2-1 gene was paired with TRAJ33/20/21 genes in a significant percentage of the cells (Supplemental Table 7). This TCR pairing was reminiscent of mucosal associated invariant T (MAIT) cells, a unique innate-like T cell subset, activated by conserved bacterial ligands presented by the invariant MHC MR1 molecule in host defense against bacterial and viral infections (36, 37) that can have a deleterious function in autoimmune diseases (38–40), especially when CD25 is upregulated on the cells (41). Although single-cell analysis of differentially expressed genes in the MAIT cell cluster (cluster 6 in Supplemental Figure 3B) showed limited differences between Treg-T1D and TILT samples at day 0, by day 7, after the first course of IL-2, activation markers such as PRF1, CD69, GZMA, CXCR4, and killer cell lectin like receptor B1 were upregulated in the MAIT population in the circulation of TILT patients (Figure 5E). Those results suggest that ld-IL-2 selectively promoted expansion of activated NK and MAIT cell populations in the circulation of T1D patients treated with ld-IL-2.

### Changes of CD8 population after Treg and IL-2 treatment.

**Figure 6:**
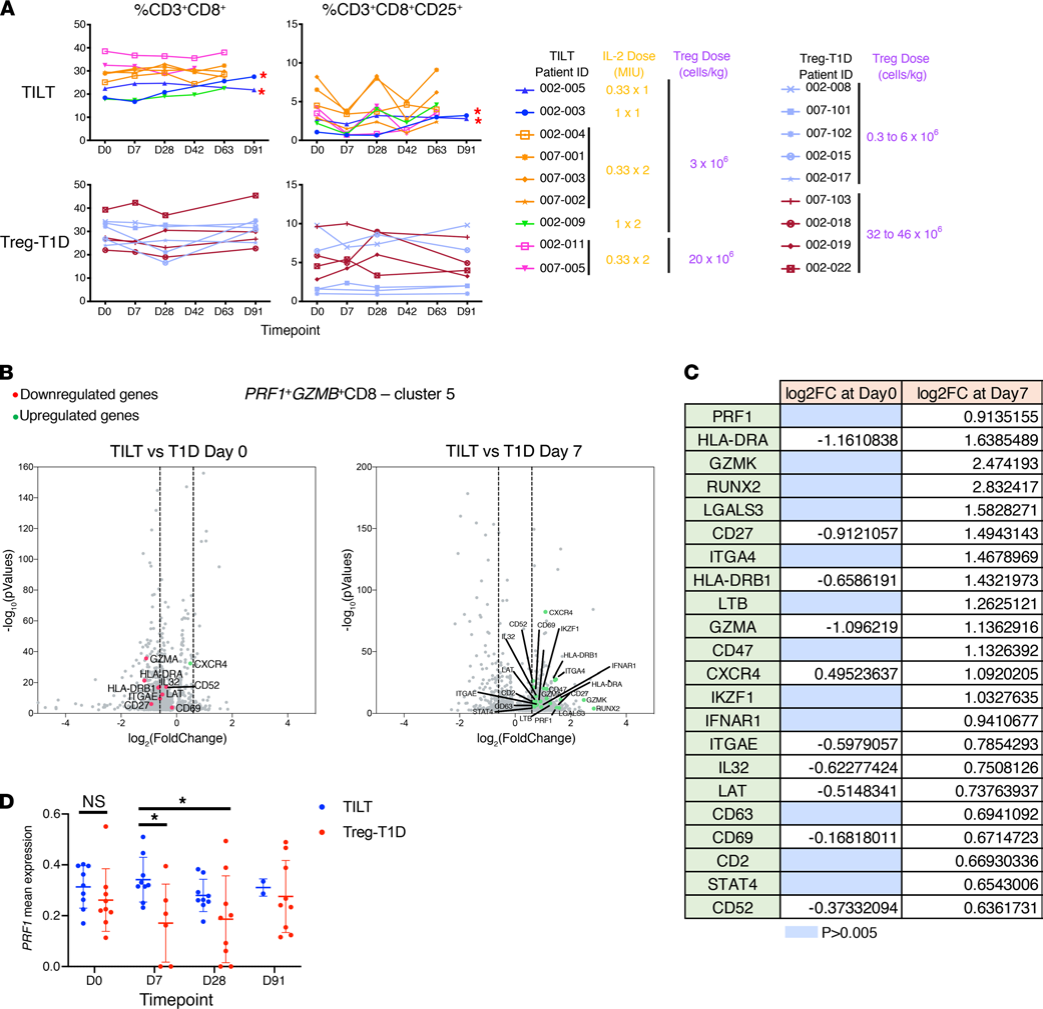
Low-dose IL-2 treatment promotes a cytotoxic phenotype in the CD8^+^ T cell subset. (**A**) Flow cytometry analysis shows the percentage of total CD3^+^CD8^+^ T cells (left column) and CD8^+^CD25^+^ T cells (right column) at the collected time points. Upper graphs represent patients from the TILT trials, and lower graphs represent patients from the Treg-T1D trial. The table indicates the dosage of IL-2 and Tregs for each patient. (**B**) Single-cell RNA-Seq data were analyzed by Scanpy package. Volcano plots represent differential gene expression analysis of the *PRF1^+^GZMB^+^*CD8 T cell compartment (Supplemental Figure 2, cluster 5) from TILT and Treg-T1D patients at day 0 (left volcano plot) and day 7 (right volcano plot). Downregulated (red dots) and upregulated genes (green dots) are indicated in log2FC with a *P* < 0.005. Gene expressions with a *P* values greater than 0.005 were filtered out. Vertical dashed lines represent thresholds of log2FC of –0.6 and 0.6 corresponding to a fold change of 1.5 times. Table indicates the log2FC values of the indicated genes. Blue cells indicate non-significant genes filtered out due to a *P* > 0.005. (**C**) Table indicates the log2FC values of the indicated genes. Blue cells indicate nonsignificant genes filtered out due to a *P* > 0.005. (**D**) Dot plot shows *PRF1* mRNA mean expression over time in *PRF1^+^GZMB^+^*CD8*^+^* T cell cluster in TILT and Treg-T1D trial patients. Asterisks indicate significance relative to the control group determined by 1-way ANOVA. *P < 0.05.

Flow cytometry analysis of the PBMCs from Treg and ld-IL-2–treated patients showed that the overall percentage of CD3^+^CD8^+^ remained unchanged (Figure 6A), similar to what has been seen in other clinical studies. However, the percentage of CD3^+^CD8^+^CD25^+^ subset contracted after the first or second course of ld-IL-2. This was followed by a rapid recovery in the majority of the patients and, in some cases, exceeded the day 0 baseline when examined at day 63. These variations may be the consequence of the expansion of Treg population negatively regulating the expansion of the CD3^+^CD8^+^CD25^+^ subset or, alternatively, extravasation of this activated subset of cells from the peripheral blood compartment.

Further analysis by single-cell RNA-Seq analysis supported the finding that selected activated CD8^+^ T cell subsets were altered dynamically by the ld-IL-2 plus Treg therapy. Specifically, there were significant changes in the gene expression profile of an activated CD8^+^ T cell subset expressing PRF1 and GZMB, 2 major cytotoxic proteins expressed by CD8^+^ T cells after activation (cluster 5, Supplemental Figure 3). Analysis of differentially expressed genes between Treg-T1D and TILT samples showed minor differences within the PRF1^+^GZMB^+^CD8^+^ T cells at day 0, while activation markers such as PRF1, CD69, and HLA-DRA were upregulated in the TILT patient samples by day 7, after the first course of IL-2 (Figure 6, B and C). Longitudinal analysis showed that PRF1 mean expression in cluster 5 was higher in TILT trial patients compared with Treg-T1D trial patients at day 7 after the first course of IL-2 (Figure 6D). These results suggest that the ld-IL-2 treatment expanded a preexisting population of PRF1^+^GZMB^+^CD8^+^ T cells in a subset of the patients. It is important to note that we have only assessed circulating cells. There may be activated cells in the inflamed tissue that may be mobilized as a consequence of the treatment, resulting in an increase in activated cells in the circulation.

**Figure 7:**
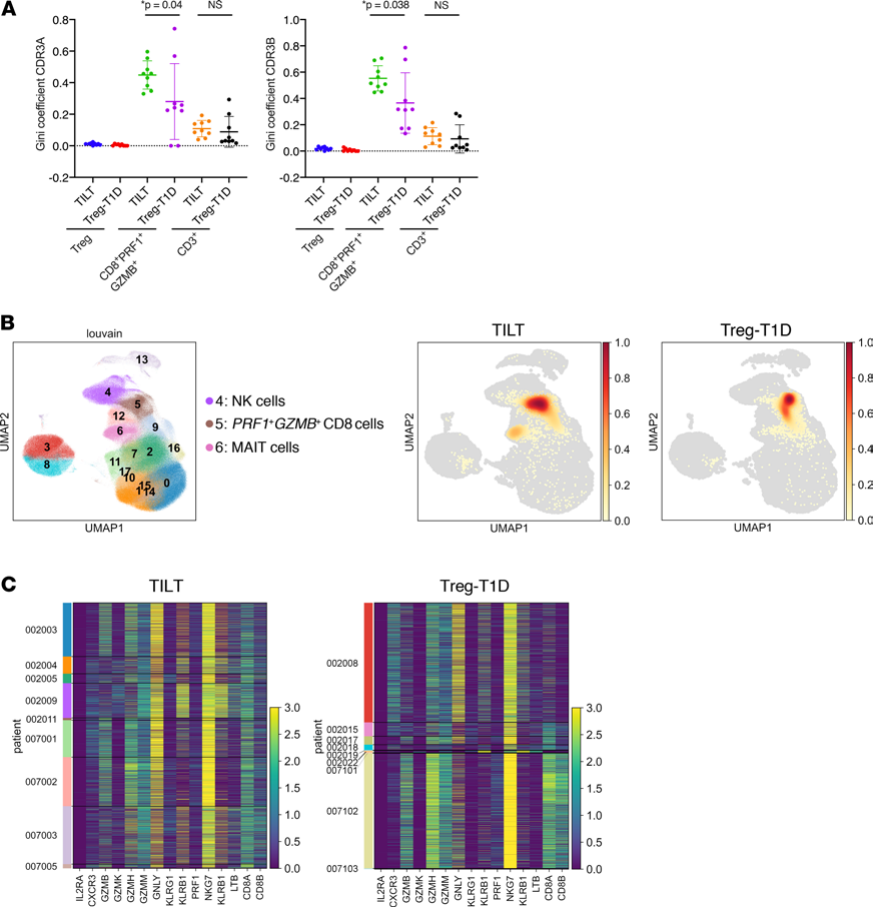
Low-dose IL-2 increases clonal expansion of the *PRF1^+^GZMB^+^*CD8^+^ T cell compartment. (**A**) Clonal diversity from the Treg, *PRF1*^+^*GZMB*^+^CD8 T cell, and total *CD3*^+^ populations was evaluated by the calculation of the Gini index. Dot plots show values of Gini index for each patient from the TILT and the Treg-T1D trial. Unpaired 2-tailed *t* tests were performed in order to assess statistical significance. (**B**) Left UMAP plot represents clusters of immune cells identified in Supplemental Figure 2. Density plots on the right represent mapping of TCR clones expanded more than 30 times in each clinical trial group. (**C**) Heatmaps represent cytotoxicity and activation markers gene expression (log normalized) of all the cells expressing expanded TCRs. Left *y* axis links the patient to the depicted expanded TCRs.

In order to assess whether the activated PRF1^+^GZMB^+^CD8^+^ T cells represented the expansion of a clonal population, we evaluated the sequences of TCRα and β chains. Analysis of the Gini coefficient of individual T cell subsets for the TCRα chains (left) and the TCRβ chain (right) was determined (Figure 7A). The Gini coefficient is a measure of statistical dispersion intended to represent the distribution of the clonal diversity. As expected, the Gini index value was close to null in the Treg compartment for patients from the 2 trials, reflecting the high diversity of Treg TCRs with no clonal expansion. However, there were substantial changes in diversity of TCR usage in the PRF1^+^GZMB^+^CD8^+^ T cell compartment. Dot plots shown for both TCR chains demonstrated an increased Gini index in the Treg-T1D patients, indicating a preexistent clonal expansion of this T cell subset. Moreover, ld-IL-2 further enhanced the clonal expansion of this population, consistent with a role for IL-2 in the expansion of activated PRF1^+^GZMB^+^CD8^+^ T cells. In this regard, the Gini index calculation for TCRα and TCRβ for the whole CD3^+^ T cell population was unaltered after ld-IL-2 treatment or in samples from the Treg-T1D trials, suggesting that effects of ld-IL-2 treatment were manifested in the PRF1^+^GZMB^+^CD8^+^ T cell subset selectively (Figure 7A). The top CDR3 α and β sequences and frequencies from the PRF1^+^GZMB^+^CD8^+^ T cell population from the TILT patients are shown in the Supplemental Table 6. TCR sequences observed more than 30 times in the single-cell sequencing analyses mostly mapped within the PRF1^+^GZMB^+^CD8^+^ cluster 5 in both clinical studies (Figure 7B, Treg-T1D and TILT UMAPs). The cells from which these TCRs were derived (Figure 7C) exhibited very similar gene expression profiles for activation markers and cytotoxic genes in the TILT and Treg-T1D patients, confirming that a similar PRF1^+^GZMB^+^CD8^+^ T cell subset was present in both patient groups but selectively enriched in the ld-IL-2–treated TILT patients.

## Discussion

The conduct of the TILT trial was designed to test for the safety of the combination of Tregs and changes in molecular and cellular biomarkers that could be used to assess the clinical impact of T1D disease progression. There was no evidence that the combined therapy led to preservation of insulin production. Moreover, there were limited effects on the proportion of circulating adoptively transferred polyclonal Tregs (PolyTregs), and we were unable to determine if there were functional changes in the cells, as the cells could not be analyzed on a per-cell level. However, the longitudinal study confirmed that the ld-IL-2 treatment increased the number of endogenous Tregs in the peripheral blood as previously reported (20, 42–45). Moreover, there were transient changes in the distribution of the adoptively transferred Treg product, something underappreciated in previous studies of ld-IL-2 treatment, where individual Treg populations could not be tracked effectively. Importantly, the treatment led to the expansion of PRF1- and GZMB-expressing CD8^+^ T, MAIT, and NK cells and clonal expansion of a subset of the activated CD8^+^ T cells. This effect was likely due to expansion of preexisting activated immune cells, including a subset of activated CD8^+^ T cells expressing high levels of the IL-2 receptor (CD25, CD122, CD132) complex cells in response to the exogenously administered IL-2. These results support the hypothesis that ld-IL-2 expands exogenously administered Tregs but also indicates how the same dose of ld-IL-2 can expand non-Tregs with increased expression of activation/cytotoxic genes in several immune cell subsets, potentially shifting the immune balance toward activation rather than tolerance. These results have important implications for the use of a combination of ld-IL-2 and Tregs for the treatment of autoimmune disease.

Many studies of IL-2 and CD25 deficiency have demonstrated that IL-2 is critical for the maintenance of the survival and the function of Treg cells (20, 42–45). In adoptive Treg transfer therapies, the survival and function of infused Tregs is the key to their potential efficiency in the prevention of T1D progression. In a polyclonal Treg therapy trial, Marek-Trzonkowska et al. showed that infusing up to 2 doses of Tregs at 30 × 10^6^/kg in young individuals led to a decrease of IL-2 in the serum of T1D patients after Treg infusion (46). In addition, in a previous Treg ACT trial, we showed a decrease of the adoptively transferred ex vivo expanded Treg populations 2 weeks after ACT, which may have reflected lack of access to IL-2 in the periphery (12). Therefore, we hypothesized that the combination of ld-IL-2 and Tregs might result in a more robust survival and function of the adoptively transferred polyclonal Tregs. In fact, multiple studies have examined the use of ld-IL-2 therapy to boost Treg cell survival and function for the treatment of autoimmunity and graft-versus-host disease (GvHD) (21, 30, 34, 47, 48). One of the earliest studies testing the effects of ld-IL-2 treatment was performed after hematopoietic stem cell transplantation. The investigators showed that treatment with ld-IL-2 increased early Treg expansion and suppressed acute and chronic GvHD (21, 22). Moreover, in 2011, Saadoun et al. showed that ld-IL-2 treatment could reduce hepatitis C virus–induced (HCV-induced) vasculitis in a majority of patients (34). Several T1D clinical trials using ld-IL-2 have been conducted, including a phase I/II trial IL-2 dose-defining study of 24 patients with T1D. In this study, patients were treated with 0.33 M to 3 M IU daily, and the treatment was shown to be well tolerated and led to an increase in the percentage of Tregs (28), although it should be pointed out that Treg percentages went back to pretreatment levels after discontinuation of the therapy. Importantly, in a study that tested a higher dose of IL-2 combined with rapamycin, an increased and persistent ability of IL-2 to induce STAT5 phosphorylation, a defect found in endogenous Tregs isolated from patients with T1D, was observed (49).

The gene expression profile of endogenous Tregs was assessed in order to understand whether ld-IL-2 could have affected the phenotype and function of the Treg population in patients with T1D. In all the TILT patients, CyTOF, flow cytometry, and single-cell RNA-Seq data showed that the whole population of Tregs exhibited upregulated activation markers, including CD25, GITR, CD27, HLA-DRA, and GARP, after ld-IL-2 treatment. Moreover, an increase in Treg memory phenotype and secondary lymph node homing marker expression in the whole Treg population was observed, suggesting that there was dynamic circulation of Tregs between tissues and the peripheral blood. Taken together those data corroborate previous studies showing that ld-IL-2 treatment resulted in an expansion of Tregs with an activated and memory phenotype (30, 35, 50). In parallel, the overall increase in the percentage of adoptively transferred Tregs in the peripheral blood, as shown by the ^2^H tracking, suggested that the ld-IL-2 treatment enhances survival and/or redistribution of Tregs in the circulation. In the latter case, the ld-IL-2 may have activated ACT Tregs, which then redistributed from the tissue where they had migrated after the transfer. Similar results were observed in the single dose of subcutaneous aldesleukin in the Adaptive Study of IL-2 Dose on Regulatory T Cells in type 1 Diabetes trial (DILT1D), where Todd et al. showed the early altered trafficking of the Treg population (51). However, the limitation of using ^2^H labeling and subsequent mass spectrophotometry analyses is that the labeling method does not allow the determination of proliferative activity as the label dilutes during this process, allowing only a summation of total percentage of ^2^H-enriched DNA as a percentage of total Tregs. In summary, the impact of ld-IL-2 on the endogenous and infused Tregs populations in the TILT study was similar as compared with other clinical trials treating patients with T1D with ld-IL-2, and no evidence links those changes to a less functional Treg compartment.

In the TILT study, we observed that some patients treated with the ld-IL-2 plus PolyTregs showed a transient downregulation of both NK and CD25^+^CD8^+^ population followed by a recovery phase. Other patients showed increased percentages of activated GZMB^+^ NK, PRF1^+^ MAIT, and PRF1^+^GZMB^+^CD8^+^ T cell subsets. Several studies in healthy donors and GvHD showed that ld-IL-2 induced proliferation of non-Treg populations such as NK and CD8^+^ T cells (29, 35). In T1D, Todd et al. reported in an IL-2 clinical trial that a single dose ranging from 0.04 to 1.5 MIU/m^2^ body surface area induced transient downregulation of NK cells’ percentage in circulation at 90 minutes followed by an expansion of the population above baseline in an IL-2 dose-dependent manner (51). These results and others (35, 52) suggest that ld-IL-2 treatment affects the homeostasis of NK and activated CD8^+^ T cells, raising a concern about the limited dosing window with this cytokine. In another dose-defining study in T1D, Rosenzwajg et al. reported a transient induction of CD8^+^ T cells at 3 MIU/d, and the NK gene expression signature was upregulated. The doses used in the TILT study were similar to those reported (0.33 to 1 MIU/d for 5 consecutive days) (35). Most recently, the same group tested an ld-IL-2 in children with T1D within 3 months of diagnosis (52). The authors showed by immune cytometry no differences in the CD8 and NK population between IL-2–treated and placebo groups. However, the dosage used was significantly different from the TILT trial as 0.125, 0.250, or 0.500 MIU/m^2^ was given daily for a 5-day course and then every 2 weeks for 1 year.

A number of factors, including the dosage scheme, amount of IL-2, regimen of ld-IL-2 administration, or length of administration, as well as disease-related factors such as time after diagnosis or age, might also affect the outcomes with IL-2 administration. Moreover, it has been shown that genetic variants of the enhancer region of IL-2RA could affect activation of the gene (53). In the present study, there was a positive correlation between IL-2–dependent NK/Treg proliferation, suggesting that the effectiveness of ld-IL-2 may depend on responsiveness and sensitivity of IL-2 activation/regulation pathways in the Treg and the cytotoxic cell populations. In addition, differences in the basal level of percentage of GZMB^+^ NK Treg-T1D and TILT patients could account for the effects of IL-2 administration on the expansion of non-Treg cells. It is possible that the presence of preexisting populations of activated cells, either due to the remitting/relapsing nature of the disease or viral or other infections in the patients concurrent with ld-IL-2 administration, could impact effector cell expansion. In this regard, it should be noted that 2 patients in the Treg-T1D trial showed expanded activated Tregs.

The characterization of specificities of the expanded PRF1^+^GZMB^+^CD8^+^ T cells for self-antigen and/or viruses such as CMV or EBV would be informative in order to determine if IL-2 targets a functional autoimmune subset of the adaptive immune cell population. Age and environmental exposure play a major role in shaping TCR repertoires in each individual; those factors may therefore play an important part in the response of the cytotoxic CD8 compartment to Proleukin. Taken together these data suggest that the individual’s TCR repertoire of cytotoxic/activated immune cell populations could be targeted by ld-IL-2 treatment. Profiling of the basal cytotoxic immune cells in young children, healthy individuals, or other autoimmune disease patients may bring more insight to cellular mechanisms involved in the onset and development of T1D, as well as the impact of Proleukin and other immunotherapies within diverse groups of individuals.

There were limitations of our clinical study. Although we did not detect a significant improvement or even maintenance of insulin secretion in the participants studied when compared with placebo-treated patients from 2 other clinical trials, the number of patients that were studied was small and lacked clear statistical power to declare a correlation between IL-2–induced activation of cytotoxic cells and the inability of the combined therapy to maintain or improve C-peptide level. The concern about dramatic reduction in C-peptide production at 28 days led the Data and Safety Monitoring Board (DSMB) to recommend altering the IL-2 dose and ultimately led to termination of the study because of the low likelihood that clinical benefit could be achieved with the Treg plus ld-IL-2 combination. This decision was based on current information available to the DSMB and based on incomplete data on the impact of T1D on C-peptide production in short intervals of time. Only through retrospective comparisons with a placebo group identified through access to data in other clinical trials including patients who were age matched was it determined that many patients can experience dramatic reduction in C-peptide production in this limited time frame. However, even given these additional comparative data, it was clear that the combination therapy did not significantly preserve or improve C-peptide production in the treated individuals.

In conclusion, the off-target effect of ld-IL-2 stresses the need to develop immunotherapy molecules targeting the Treg population more specifically. We and others have developed reagents such as antibodies, pegylated IL-2, engineered or even mutant IL-2 that can expand Tregs but not NK or CD8^+^ T cells (54–57). These studies show the versatility of IL-2 and lay the foundations for design of a more specific cytokine in the treatment of T1D. Furthermore, a better understanding of the endogenous cytotoxic profiles in patients with T1D and other autoimmune diseases would be also valuable in order to anticipate the effect of IL-2 treatment in future clinical trials.

## Methods

### Participants.

This study enrolled men and women diagnosed with T1D according to the American Diabetes Association standard criteria within 3 to 24 months of screening who were 18 to 45 years of age with peak C-peptide greater than 0.2 pmol/mL (0.6 ng/mL) during MMTT challenge and who were positive for at least 1 of the following antibodies: ICA 512-antibody (IA-2ic), glutamate decarboxylase (GAD), insulin, and zinc transporter 8 (ZnT8). Patients also had to have adequate venous access to support draw of 400 mL of whole blood and infusion of investigational therapy. They were determined to be ineligible if they had hemoglobin < 10.0 g/dL; leukocytes < 3000/mL; neutrophils < 1500/mL; lymphocytes < 800/mL; platelets < 100,000/mL; Tregs < 10/mL; evidence of active infection (HIV-1/HIV-2, hepatitis B, hepatitis C, EBV or CMV genomes, or positive purified protein derivative skin test); chronic use of systemic glucocorticoids or other immunosuppressive agents or biologic immunomodulators within 6 months before study entry; history of malignancy except adequately treated basal cell carcinoma; or any chronic illness or previous treatment that, in the opinion of the investigator, should preclude participation in the trial. Pregnant or breastfeeding women were excluded from the study, as well as any woman who was unwilling to use a reliable and effective form of contraception for 2 years after Treg dosing, and any man who was unwilling to use a reliable and effective form of contraception for 3 months after Treg dosing.

### Administration and follow-up.

Results of blood chemistries and hematology were reviewed, and a history of any recent illness or fever was obtained before infusion of the cells. Patients received premedication with acetaminophen and diphenhydramine. PolyTregs were manufactured under cGMP conditions from autologous participant whole blood, as previously described (12). PolyTregs were infused fresh after a 14-day ex vivo expansion via a peripheral intravenous line over 10 to 30 minutes. Vital signs were taken before infusion and 15, 30, 60, 120, and 240 minutes postinfusion. Participants were discharged after being monitored at the clinical research unit for 4 hours postinfusion. Participants were seen for follow-up assessments on days 1, 3, 7, 14, 28, 38, 42, 49, 63, and 91 after infusion, then every 13 weeks up to 1 year postinfusion, and then every 26 weeks up to 2 years postinfusion. Telephone monitoring for adverse events continued every 26 weeks up to 3 years after infusion.

### IL-2 administration.

Each course (5 consecutive days) of IL-2 was prepared by, and dispensed from, the investigational pharmacy at each clinical site for administration by subcutaneous injection in the thigh at days 3–7 and 38–42 (if applicable). Clinical personnel at the research site administered the first injection of each 5-day course of IL-2. Self-administration by participants was allowed for subsequent IL-2 injections. An administration diary was provided to record the date, time, and injection site for each administration. The cell infusions were well tolerated based on safety criteria outlined in the protocol. There was a precipitous decline of C-peptide (<50% of the baseline C-peptide response at day 28) in the first patient (patient 002-003) enrolled in the trial. Thus, the second course of IL-2 was withheld based on a mandated stopping rule and DSMB review. The rapid loss of C-peptide production was observed in 2 additional participants (002-003 and 002-005), who were administered 1 × 10^6^ and 0.33 × 10^6^ U/d 5 times, respectively (Supplemental Table 1). These results led to a modification of the protocol with additional participants in the second cohort receiving 2 courses of IL-2 of 0.33 × 10^6^ U/d × 5 each.

### Phenotypic analysis of expanded Treg populations and peripheral blood samples.

Frozen PBMCs were thawed in 10 mL X-VIVO media, spun down, and resuspended in 5 mL of X-VIVO media. Each sample was counted and distributed into 3 aliquots and processed for flow cytometry analysis, CyTOF, and 10x Genomics analysis. Phenotypic and functional analyses showed that the percentage and level of expression of FOXP3^+^ in expanded Tregs before infusion was similar in this trial as compared with the previous Treg-T1D study (Supplemental Figure 4). Moreover, the expanded population showed high levels of CD25 expression as well as a highly demethylated Treg-specific demethylated region (TSDR) locus consistent with a bona fide Treg population (Supplemental Figure 4).

### Flow cytometry.

A total of 1 × 10^6^ PBMCs were washed in FACS buffer (phosphate-buffered saline + 1% FBS) and distributed into 96-well plates. Cell were stained in FACS buffer containing 1/500 of LIVE/DEAD blue reagent (Invitrogen) and incubated at room temperature for 30 minutes protected from light. After 1 wash with FACS buffer, cells were incubated with human Fc block reagent for 5 minutes, stained with cell surface fluorochrome-conjugated anti-human CD19, CD45RA, HLA-DR, CD8, CD127, CD27, CD4, CRO, CD40, CD56, CD38, CD3, CD14, and CD25 for 30 minutes on ice. After 2 washes in FACS buffer, cells were analyzed by BD LSRII flow cytometer. All data analysis was performed using FlowJo software. Antibody panel is listed in Supplemental Data File 2.

### CyTOF.

Cryopreserved cells were thawed and placed in 10 mL of 10% FBS-DMEM and pelleted, resuspended in 2 mL of the same media, and counted, and 1 × 10^6^ cells were removed for CyTOF staining. Cells were centrifuged and resuspended in 0.5 mL PBS in 15 mL conical tubes, and an equal volume of 1:5000 diluted cisplatin was added for a final concentration of 5 μM (Fluidigm catalog 201198) and incubated at room temperature (RT) for 5 minutes. Cisplatin reaction was quenched by adding 14 mL CSM (0.5% BSA, 2 mM EDTA in PBS) to each tube and pelleted. Cells were resuspended in 50 μL of FcBlock (Miltenyi Biotec catalog 130-059-901), diluted to manufacturer’s recommendations for 5 minutes. Mass cytometry antibodies were previously titrated using cryopreserved peripheral blood controls to achieve optimal signal-to-noise ratios. After blocking, 50 μL of cell surface cocktail was added and incubated on ice for an additional 40 minutes. Antibodies were washed with CSM and pelleted. Cells were resuspended in residual volume after aspiration of CSM wash, and 200 μL of fix/perm buffer (eBioscience catalog 88-8824-00) was added to each sample and incubated for 30 minutes at RT. Subsequently, 1 mL of diluted eBioscience Perm Buffer (catalog 00-8333-56) was added and centrifuged at 400*g* for 7 minutes. These centrifuge conditions were used for all subsequent steps. The pellets were resuspended in 50 μL intracellular antibodies diluted with the Perm Buffer and incubated on a shaker for 1 hour at RT. One mL of diluted Perm Buffer was added to each tube and centrifuged. Cells were further washed with 14 mL CSM and pelleted. Pellet was resuspended in 1 mL of 3.2% paraformaldehyde, 0.02% Saponin (MilliporeSigma catalog 47036), and 100 nM Ir (iridium) used for identifying nucleated cells (Fluidigm catalog 201192B)/PBS and stored at 4^o^C. Prior to running the samples on the CyTOF machine, 10 μL of 20-plex Pd (palladium) (15 μM each isotope, UCSF flow core) barcode was added to each sample and incubated at RT for 20 minutes. A total of 14 mL of CSM was added to each tube to sequester the remaining free Pd isotopes and the tubes were centrifuged. Each barcoded sample was resuspended in 0.5 mL PBS and pooled into a single 15 mL conical tubes and centrifuged. Pooled cells were resuspended in MilliQ water (MilliporeSigma) and centrifuged at 400*g* for 10 minutes. Cells were resuspended in water containing 1:10 diluted Eq beads (Fluidigm catalog 2010778) and adjusted to a concentration of 1.2 × 10^6^/mL and run on the Helios CyTOF at approximately 500 cells per second at the UCSF flow core. Antibody panel is listed in Supplemental Data File 2. FCS files acquired on Helios CyTOF at approximately 500 cells per second at the UCSF flow core are available on request.

Data normalization was performed as previously described (58). All mass cytometry files were normalized together using the mass cytometry data normalization algorithm (59), which uses the intensity values of a sliding window of these bead standards to correct for instrument fluctuations over time and between samples. After data collection, each condition was deconvoluted using a single-cell debarcoding algorithm (60). After normalization and debarcoding of files, singlets were gated by event length and DNA. Live cells were identified by cisplatin-negative cells. All positive and negative populations and antibody-staining concentrations were determined by titration on positive and negative control cell populations. After debarcoding and normalization of CyTOF data, cell populations were gated manually in CellEngine. Populations were then exported for analysis in R. Marker expression values were then arsinh-transformed with a cofactor of 5, and then expression values from all cells from a trial and time point were plotted as a violin plot using ggplot2. The results are plotted into 2 separated batches (1 and 2) due to batch effect affecting the comparison of the samples within the same analysis pipeline (batches layout of the samples, Supplemental Table 3).

### 10x Genomics.

For 10x Genomics single-cell RNA-Seq, we first used FACS to isolate living cells (CD45^+^ and LIVE/DEAD blue) and then implemented the Chromium Single Cell V(D)J Enriched and 5*′* Gene Expression library generation and sequencing under the guidance of the official instruction manual. Cells were loaded onto a Chromium Next GEM chip G. Cells were lysed for reverse transcription and cDNA amplification in the Chromium Controller (10x Genomics). Then 76 samples were pooled into 5 samples, and 3 duplicate wells were run for each sample pool. Full-length cDNA along with cell barcode identifiers were PCR amplified, and sequencing libraries were prepared and normalized. The constructed library was sequenced on NovaSeq S4 flow cell (Illumina). Libraries’ preparation and sequencing were performed at the institute for human genetics at the Parnassus campus of UCSF. Cell Ranger Single-Cell Software Suite (version 3.1.0, 10x Genomics) was used for library demultiplexing, FASTQ file generation, read alignment, doublet filtering, barcode counting, unique molecular identifier (UMI) counting, and to generate feature-barcode matrices, determine clusters, and perform gene expression analysis. Droplet-based sequencing data were aligned and quantified against the GRCh38 human reference genome. Quality of cells in each demultiplexed sample was then assessed based on 3 metrics: the number of total UMI counts per cell (library size), the number of detected genes per cell, and the proportion of mitochondrial gene counts. After quality control metric filtering, a total of 464,348 cells were retained for downstream analysis.

Using Scanpy (61), we performed BBKNN batch correction and principal components analysis on the gene expression matrix, and we then built a nearest neighbor graph to calculate a UMAP and find clusters via the louvain algorithm. Clusters were identified based on the expression of canonical marker genes. Differential gene expressions were generated using Scanpy, and volcano plots depicting gene expression changes in distinct immune cell subsets were generated using GraphPad Prism 6.0 software.

### Single-cell TCR-Seq data processing and RNA-Seq integration.

Data from 10x Genomics single-cell VDJ sequencing were aligned and quantified using the Cell Ranger Single-Cell Software Suite (version 3.1.0) against the GRCh38 human VDJ reference genome. Resultant filtered annotated contigs were analyzed and concatenated to the 10x single-cell gene expression data in python.

Frequency of each clonotype was calculated in python and Gini coefficient was calculated using Olivia Guest, Gini, and Calculate the Gini coefficient of a numpy array, from GitHub repository, https://github.com/oliviaguest/gini The Gini coefficient measures the inequality among values of a frequency distribution. A Gini score equals 0 when each element of the population has the same frequency. A high Gini score, closer to 1, suggests clonotype enrichment.

### Demuxlet pipeline analysis.

Samples from each patient were genotyped by Infinium Omni Express Exome array (Illumina). Resulting VCF genotype files and single-cell RNA-Seq BAM files were used to run Demuxlet (31) with default parameters. Demultiplexing results were largely concordant with freemuxlet (https://github.com/statgen/popscle/). Subsequently, the multiplexed single-cell RNA-Seq and single-cell TCR-Seq data were filtered according to a singlet white list extracted from the demuxlet.best output file.

### TSDR methylation assay.

Genomic DNA from 1 × 10^6^ expanded Tregs was analyzed by Epiontis GmbH according to established protocol. Percentages of demethylated TSDR were calculated as follows: (mean copy numbers of unmethylated DNA/[mean copy numbers of unmethylated DNA + copy numbers of methylated DNA]) × 100. For Tregs from women, the percentages calculated above were multiplied by 2 to correct for X chromosome inactivation.

### Treg ^2^H tracking.

During the 14-day clinical expansion period, the ^2^H_2_ label from [6,6-^2^H_2_]glucose in the X-VIVO 15 culture medium was incorporated into the DNA of replicating polyclonal CD4^+^CD127^lo/−^CD25^+^ Tregs as previously described (42). Initial qualifying experiments showed no differences in fold expansions, phenotype, or percentage TSDR demethylation between Tregs grown in X-VIVO 15 and Tregs grown in [^2^H_2_]glucose-containing X-VIVO 15. Functional suppression assay results showed similar inhibition between Tregs expanded in either type of medium, and cultures were free from bacteria, fungi, mycoplasma, or endotoxin contaminants. Mass spectrometry analyses showed that Tregs expanded in X-VIVO 15 with [6,6-^2^H_2_]glucose in the medium at 100% enrichment were approximately 60% enriched for ^2^H_2_ in the deoxyribose moiety of purine deoxyribonucleotides isolated from DNA, which is the theoretical maximum deuterium enrichment observed in deoxyribose in fully replaced cells that divided in the presence of [6,6-^2^H_2_]glucose (42, 43). This 60% enrichment level was consistently observed in all 7 preparations in this clinical study.

After infusion of the labeled Tregs, peripheral blood was collected from the study participants, and PBMCs were sorted into the Treg population (CD4^+^CD25^+^CD127^lo/–^) and 3 non-Treg populations (CD4^+^CD25^lo^CD127^hi^ CD45RO^+^, CD45RO^+^CD62L^lo^, CD45RO^+^CD62L^hi^). Deuterium enrichments were compared between the Treg population and a mixture of the 3 non-Treg populations.

### Laboratory tests.

Biochemical autoantibody titers were assayed at the Barbara Davis Center using radioimmunobinding assays, and ICA was measured at the University of Florida. C-peptide and HbA1c were measured at the Northwest Lipid Research Laboratory. Viral loads for EBV and CMV were assessed by ViraCor Laboratories. Chemistries and hematology were performed at local clinical laboratories at UCSF and Yale. Mass spectrometry analysis was performed the Department of Nutritional Sciences & Toxicology at the University of California, Berkeley.

### Data availability.

The 10x Genomics single-cell sequencing data reported in this paper have been deposited in the Gene Expression Omnibus database (Study: GSE178991 and Series record: GSE178991).

### Statistics.

Data analyses were performed using GraphPad Prism 6.0 software, and values at *P* < 0.05 were deemed significant. One-way ANOVA tests were performed in order to assess statistical significance.

### Study approval.

All participants provided written informed consent before participating in any study procedures. UCSF Institutional Review Board, San Francisco, California, USA, approved the study.

## Author contributions

JAB, SEG, KJHG, and QT were the principal investigators of the study, participated in its design, and analyzed results. JHE supervised Treg production and study design and monitoring. SD performed flow and 10x Genomics experiments and analysis; KCM performed analysis of CyTOF data; CTM performed Demuxlet analysis; WL, SJT, CMT, and WT performed CyTOF experiments; APL, WL, VN, ML, and ALP generated Treg products and performed the quality control assays; ASL is the clinical coordinator and analyzed clinical data and metabolic responses; WL, MM, and SD participated in sample preparation for flow cytometry, CyTOF, and 10x Genomics experiments; MHS advised for CyTOF analysis; CJY advised 10x Genomics experiment design and supervised Demuxlet analysis; and SD and JAB analyzed the results and wrote the article. All authors edited the report and approved the final manuscript.

## Supplementary Material

Supplemental data

Supplemental data 1

Supplemental data 2

Supplemental data 3

Trial reporting checklists

ICMJE disclosure forms

## Version 1 07/29/2021

In-Press Preview

## Version 2 09/22/2021

Electronic publication
