# Supplementary material for: The effect of low-dose IL-2 and Treg adoptive cell therapy in patients with type 1 diabetes
Source: JCI Insight. 2021 Sep 22;6(18):e147474. doi: 10.1172/jci.insight.147474 (PMC8492314; doi:10.1172/jci.insight.147474)
Supplement: Supplemental data [file jciinsight-6-147474-s153.pdf]

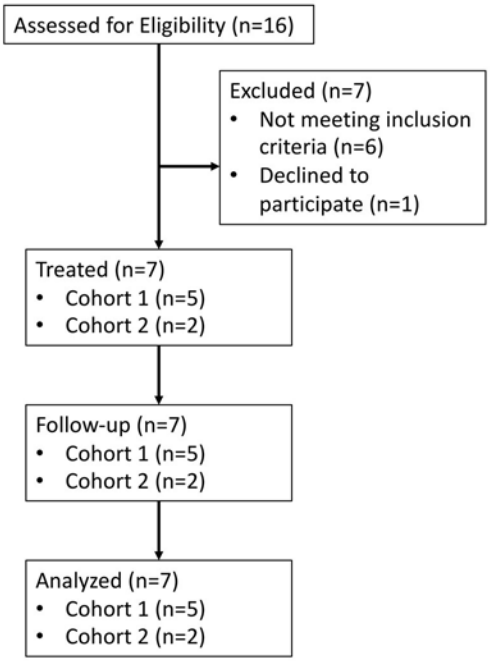

Figure S1: CONSORT diagram

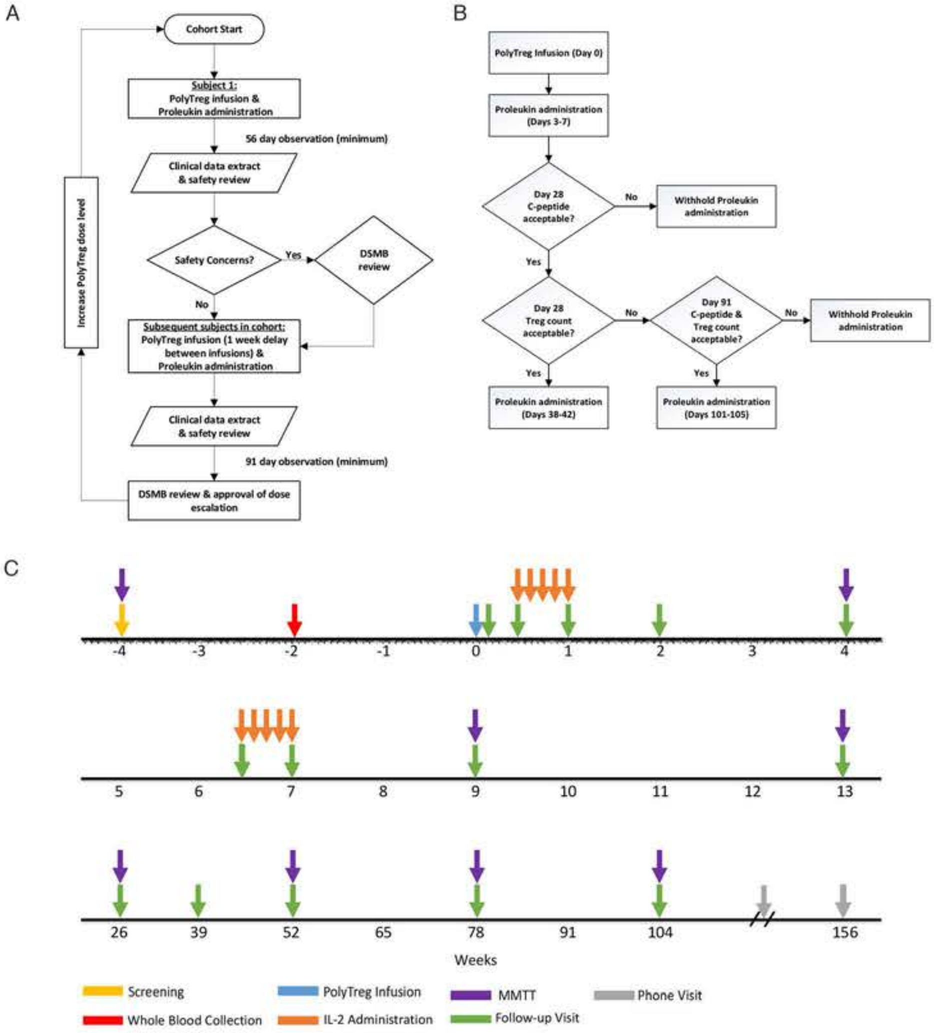

Figure S2: Study design and individual's participation plan. (A) Dose escalation plan. Subjects were enrolled in 2 cohorts with target doses of Tregs ranging from 3 to 20 × 106/kg. **(B)** Proleukin infusion plan. **(C)** Subject schedule of events. Blood (target of 400 ml) for Treg manufacturing was drawn at week –2, and Treg infusion was given on day 0. Ld-IL2 was infused during 5 consecutive days at week1 (Day 3 to Day 7) and week 7 (Day 38 to Day 42) (orange arrows). Participants were seen for follow-up assessments on days 1, 3, 7, 14, 28, 38, 42, 49, 63, and 91 after infusion (green arrows), then every 13 weeks up to 1-year post-infusion, and then every 26 weeks up to 2 years post-infusion. Telephone monitoring for adverse events continued every 26 weeks up to 3 years after infusion.

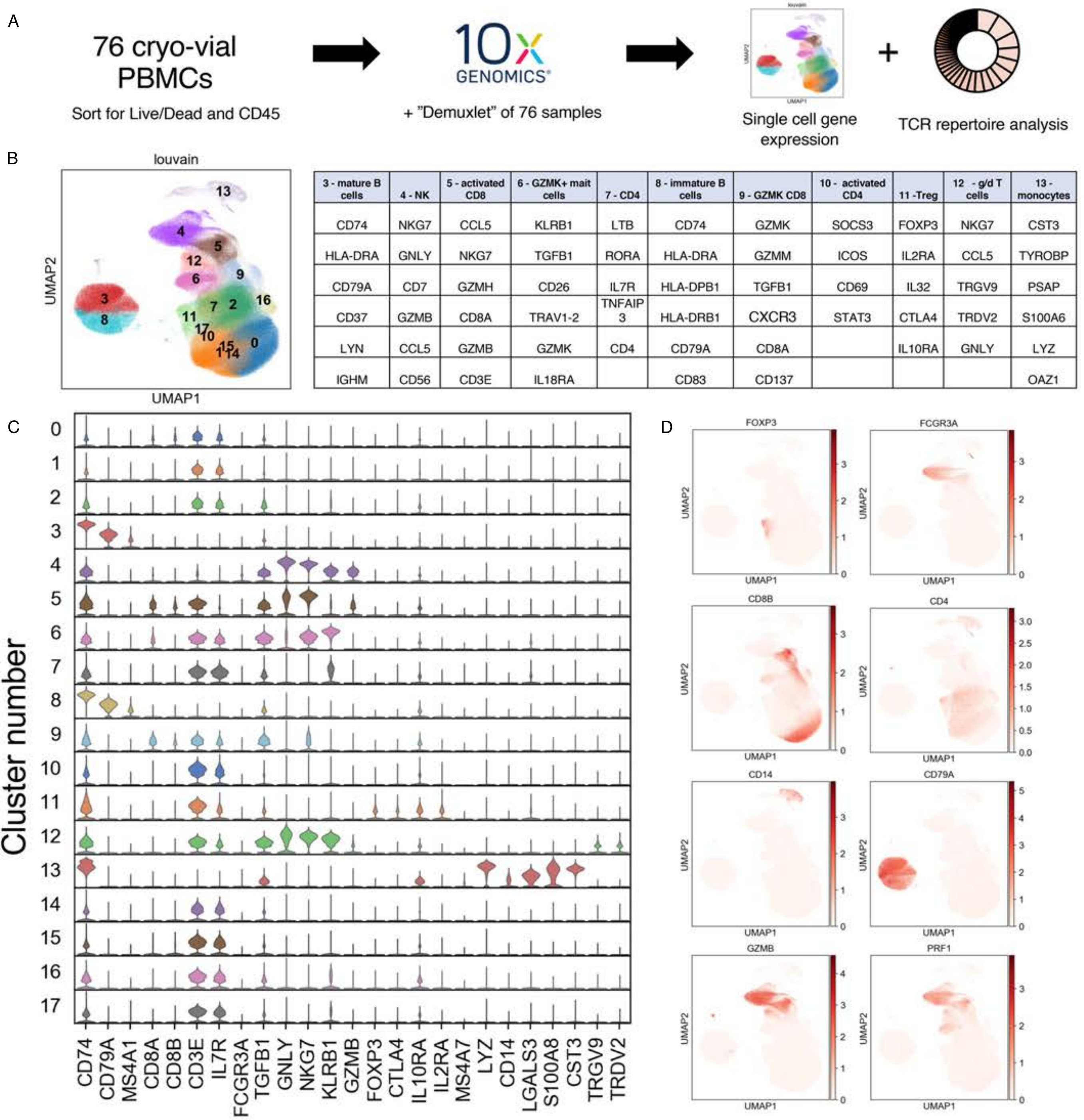

3 - mature B cells	4 - NK	5 - activated CD8	6 - GZMK+ mait cells	7 - CD4	8 - immature B cells	9 - GZMK CD8	10 - activated CD4	11 -Treg	12 - g/d T cells	13 - monocytes
CD74	NKG7	CCL5	KLRB1	LTB	CD74	GZMK	SOCS3	FOXP3	NKG7	CST3
HLA-DRA	GNLY	NKG7	TGFB1	RORA	HLA-DRA	GZMM	ICOS	IL2RA	CCL5	TYROBP
CD79A	CD7	GZMH	CD26	IL7R	HLA-DPB1	TGFB1	CD69	IL32	TRGV9	PSAP
CD37	GZMB	CD8A	TRAV1-2	TNFAIP 3	HLA-DRB1	CXCR3	STAT3	CTLA4	TRDV2	S100A6
LYN	CCL5	GZMB	GZMK	CD4	CD79A	CD8A		IL10RA	GNLY	LYZ
IGHM	CD56	CD3E	IL18RA		CD83	CD137				OAZ1

Figure S3: Gene expression profile mapping of the main immune cells present in PBMCs created by Single-Cell RNA Sequencing. (A) Schematic of the workflow used to generate the UMAP plots. PBMCs from 9 patients of the TILT study were collected at different timepoint before and after the two infusion of low-dose IL-2 and frozen down were analyzed trough the single cell RNAseq and TCR sequencing from 10X genomics technology. These samples were compared to samples 9 patients of the T1D study harvested at similar time points. A total number of 76 cryovials were pooled into 30 10X samples. The Cell ranger output raw data from were analyzed via the Demuxlet pipeline developed by the Ye Lab at UCSF in order to deconvolute the sample identity. The identified single cells datasets were preprocessed and filtered according to the Scanpy pipeline guidelines and 400,000 cells were clustered according to their gene expression profile. In parallel, single cell TCR sequences were extracted from the same dataset and matched back to the gene expression UMAP. **(B)** UMAP plots showing the clustering of PBMCs cells, colored by clusters. Each cluster were assigned to a known cell type according to their differentially expressed genes. List of the marker genes are represented in the table on the right. **(C)** Violin plots depicting the distribution of cell-type according to known immune lineage marker genes. (D) UMAP plots show the distribution of lineage markers, cytotoxic enzymes involved in immune cell migration distribution among the cell clusters.

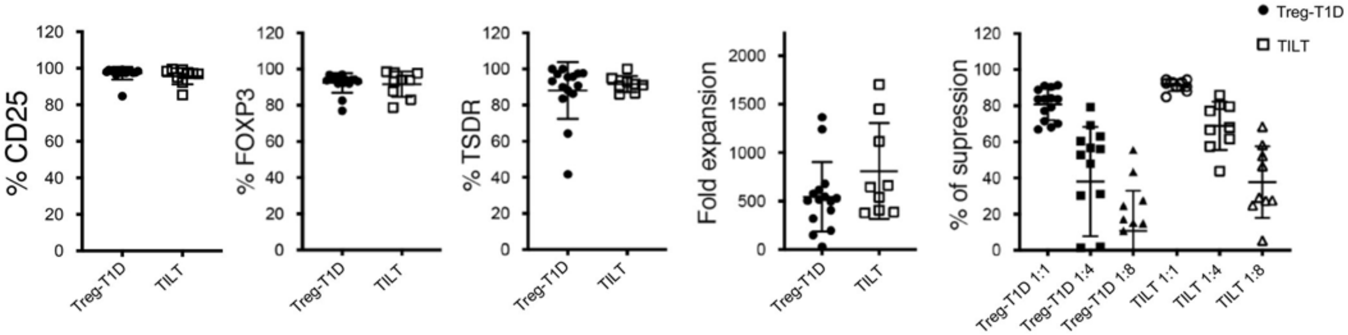

Figure S3: Phenotypic control of infused Treg products. Dot plots from left to right represent percentage of FOXP3, percentage of TSDR, in vitro fold expansion and percentage of in vitro suppression of Tconv proliferation cocultured with 1:1, 1:4, 1: 8 ratio of Treg: Tconv. Each dot plot compares the in vitro expanded Tregs from each patient of the TILT and Treg-T1D trial.

Cohort TILT	Subject ID	Age at Visit -1	Gender	Time from diagnosis at screening (Days)	HbA1c at Screening (%)	PolyTregs		Proleukin Dose 1		Proleukin Dose 2	
						Dose (Cells/kg) x10^6	Total Infused (Cells) x10^6	Dose (U/day) x10^6	Time	Dose (U/day)	Time
1	002-003	23	M	233	6.0	3	197.7	1	Days 3-7	-	-
	002-004	31	M	389	5.5	3	175.1	0.33	Days 3-7	-	-
	002-005	27	F	357	5.7	3	340.3	0.33	Days 3-7	0.33	Days 38-42
	007-001	24	F	324	5.7	3	212.2	0.33	Days 3-7	0.33	Days 41-45
	002-009	27	M	90	6.0	3	247.7	1	Days 3-7	0.33	Days 38-42
	007-002	18	M	302	5.9	3	180.5	0.33	Days 3-7	0.33	Days 38-42
	007-003	34	M	479	5.8	3	346.2	0.33	Days 3-7	0.33	Days 38-42
2	002-011	23	M	357	6.8	20	1441	0.33	Days 3-7	0.33	Days 38-42
	007-005	24	M	374	5.3	20	1252.9	0.33	Days 3-7	0.33	Days 38-42

Cohort Treg-T1D	Subject ID	Age at Visit -1	Gender	Time from diagnosis at screening (Days)	HbA1c at Screening (%)	Dose (Cells/kg) x10^6	Total Infused (Cells) x10^6
2	002-008	32	M	98	6.0	0.3	0.398
3	007-101	19	M	210	6.0	5.1	3.81
	002-015	43	M	728	5.3	3.5	3.46
	007-102	21	F	294	9.9	6.3	3.68
	002-017	24	M	203	7.1	37.6	3.31
4	002-018	43	M	189	7.0	33.0	26.8
	002-019	34	F	364	6.5	50.0	26.9
	007-103	18	F	196	6.2	43.2	29.4
	002-022	22	F	294	4.7	32.2	23.5

Table S1: Patient demographics, Treg and IL-2 dosage

	TILT patient ID								
**Timepoint**	**002-003**	**002-004**	**007-001**	**002-009**	**007-003**	**002-011**	**007-002**	**007-005**	**002-005**
**d0**	X	X	X	X	X	X	X	X	X
**d7**	X	X	X	X	X	X	X	X	X
**d28**	X	X	X	X	X	X	X	X	X
**d42**		X	X	X	X	X	X	X	
**d63**	X	X	X	X	X	X	X		
**d91**	X								X

	T1D patient ID								
**Timepoint**	**007-101**	**007-102**	**007-103**	**002-008**	**002-015**	**002-017**	**002-018**	**002-019**	**002-022**
**d0**	X	X	X	X	X	X	X	X	X
**d7**	X		X	X			X	X	X
**d28**	X	X	X	X	X	X	X	X	X
**d42**									
**d63**									
**d91**	X	X	X	X	X	X	X	X	X

Table S2: Sample layout

TILT	002-003	007-001	002-009	007-002	007-003	002-004	002-005	007-005	002-011
D0 (V0)	Batch2	Batch2	Batch2	Batch1	Batch1	Batch1	Batch1	Batch2	Batch2
D7 (V6,V3)	Batch2	Batch2	Batch2	Batch1	Batch1	Batch1	Batch1	Batch2	Batch2
D28 (V8,V6)	Batch2	Batch2	Batch2	Batch1	Batch1	Batch1	Batch1	Batch2	Batch2
D42 (13)		Batch1	Batch1	Batch1	Batch1	Batch1		Batch2	Batch2
D63 (V15)	Batch1	Batch1	Batch1	Batch1	Batch1	Batch1			Batch2
D91 (V16, V7)	Batch1						Batch1		

T1D	007-102	002-015	002-017	002-022	007-103	007-101	002-018	002-019	002-008
D0 (V0)	Batch1	Batch1	Batch1	Batch2	Batch2	Batch2	Batch2	Batch2	Batch2
D7 (V6,V3)				Batch2	Batch2	Batch2	Batch2	Batch2	Batch2
D28 (V8,V6)	Batch1	Batch1	Batch1	Batch2	Batch2	Batch2	Batch2	Batch2	Batch2
D42 (13)									
D63 (V15)									
D91 (V16, V7)	Batch1	Batch1	Batch1	Batch2	Batch2	Batch2	Batch2	Batch2	Batch2

Table S3: Sample batches for CyTOF experiments. CyTOF experiments were performed in two different batches, analyzed and presented separately due to batch effects.

TILT	002-003	002-004	007-001	002-009	007-003	002-011	007-002	007-005	002-005
d0	Batch1	Batch2	Batch1	Batch1	Batch1Batch2	Batch3	Batch1Batch2	Batch3	Batch2
d7	Batch1	Batch2	Batch1	Batch1	Batch1Batch2	Batch3	Batch1Batch2	Batch3	Batch2
d28	Batch1	Batch2	Batch1	Batch1	Batch1Batch2	Batch3	Batch1Batch2	Batch3	Batch2
d42		Batch2	Batch2	Batch2	Batch2	Batch3	Batch2	Batch3	
d63	Batch2	Batch2	Batch2	Batch2	Batch2	Batch3	Batch2		
d91	Batch2								Batch2

Treg-T1D	007-101	007-102	007-103	002-008	002-015	002-017	002-018	002-019	002-022
d0	Batch3	Batch2	Batch3	Batch3	Batch2	Batch2	Batch3	Batch3	Batch3
d7	Batch3		Batch3	Batch3			Batch3	Batch3	Batch3
d28	Batch3	Batch2	Batch3	Batch3	Batch2	Batch2	Batch3	Batch3	Batch3
d42									
d63									
d91	Batch3	Batch2	Batch3	Batch3	Batch2	Batch2	Batch3	Batch3	Batch3

Table S4: Sample batches for 10X genomics experiments.

TRAV_TRAJ_CDR3A	#
TRAV17_TRAJ57_CATTLGGGSEKLVF	3
TRAV29DV5_TRAJ58_CAASGETSGSRLTF	3
TRAV17_TRAJ13_CATDTGGYQKVTF	3
TRAV13-1_TRAJ20_CAASLNDYKLSF	2
TRAV3_TRAJ17_CAHSKAAGNKLTF	2
TRAV29DV5_TRAJ26_CAASRNYGQNFVF	2
TRAV39_TRAJ49_CAVATGTGNQFYF	2
TRAV9-2_TRAJ39_CALRGAGNMLTF	2
TRAV38-2DV8_TRAJ54_CAYRRLIQGAQKLVF	2
TRAV38-1_TRAJ57_CAFMKPLTQGGSEKLVF	2
TRAV12-1_TRAJ10_CVVRGLTGGGNKLTF	2
TRAV8-3_TRAJ49_CAVGASNQFYF	2
TRAV17_TRAJ13_CATDSGGYQKVTF	2
TRAV13-1_TRAJ43_CAASKERDMRF	2
TRAV17_TRAJ20_CATDNDYKLSF	2
TRAV26-1_TRAJ57_CIVRGSQGGSEKLVF	2
TRAV8-4_TRAJ17_CAVSKAAGNKLTF	2
TRAV9-2_TRAJ58_CALQETSGSRLTF	2
TRAV8-1_TRAJ39_CAVTDNNAGNMLTF	2
TRAV13-2_TRAJ44_CAENTGTASKLTF	2
TRAV12-1_TRAJ28_CVVNIFKAGSYQLTF	2
TRAV38-1_TRAJ49_CAFTKYTGNQFYF	2
TRAV5_TRAJ39_CAESNNAGNMLTF	2
TRAV41_TRAJ45_CAVGSGGGADGLTF	2
TRAV38-2DV8_TRAJ21_CYDFNKFYF	2
TRAV8-3_TRAJ23_CAVNGQGGKLIF	2
TRAV3_TRAJ36_CAVRLQTGANNLFF	2
TRAV5_TRAJ5_CAEGSMDTGRRALTF	2
TRAV3_TRAJ38_CAVRVNAGNNRKLIW	2
TRAV6_TRAJ4_CALTSGGYNKLIF	2
TRAV13-1_TRAJ13_CAASHSGGYQKVTF	2
TRAV29DV5_TRAJ29_CAASDSGNTPLVF	2
TRAV12-1_TRAJ37_CVVNPGSGNTGKLIF	2
TRAV12-2_TRAJ15_CAVSDQAGTALIF	2
TRAV29DV5_TRAJ49_CAASGSGDTGNQFYF	2
TRAV8-3_TRAJ58_CAVVPARTSGSRLTF	2
TRAV3_TRAJ18_CAVRDPDRGSTLGRLYF	2
TRAV27_TRAJ9_CAGVRTGGFKTIF	2
TRAV12-2_TRAJ22_CAASSGSARQLTF	2
TRAV26-2_TRAJ23_CTSYNQGGKLIF	2
TRAV30_TRAJ23_CGTELKYNQGGKLIF	2
TRAV8-3_TRAJ48_CAVGVGFGNEKLTF	2
TRAV3_TRAJ23_CAVKKGNQGGKLIF	2
TRAV12-2_TRAJ5_CARLDTGRRALTF	2
TRAV12-3_TRAJ6_CAMSAWKGASGGSYIPTF	2
TRAV13-1_TRAJ4_CAASIGGYNKLIF	2
TRAV27_TRAJ28_CAGFSGAGSYQLTF	2
TRAV12-1_TRAJ22_CVVKFSSGSARQLTF	2
TRAV21_TRAJ43_CAVRHTNNDMRF	2
TRAV4_TRAJ4_CLVGSLSGGYNKLIF	2

...

Total = 3428

TRBV_TRBJ_CDR3B	#
TRBV7-2_TRBJ2-7_CASSFDRLSYEQYF	5
TRBV14_TRBJ2-7_CASSQVIGTSYEQYF	3
TRBV5-4_TRBJ2-6_CASTNTGANVLTF	3
TRBV20-1_TRBJ2-3_CSATDRGTDTQYF	3
TRBV7-2_TRBJ1-5_CASSLERGGSQPQHF	3
TRBV27_TRBJ2-1_CASSPRISGRAYNEQFF	3
TRBV9_TRBJ2-1_CASSSRRGSYNEQFF	3
TRBV10-3_TRBJ1-1_CAISASGHPNTEAFF	3
TRBV2_TRBJ1-5_CASSMGLSNQPQHF	3
TRBV7-9_TRBJ2-7_CASSLADSREQYF	3
TRBV20-1_TRBJ2-1_CSARQTSGSLNEQFF	3
TRBV20-1_TRBJ2-1_CSARVASGSSYNEQFF	3
TRBV6-4_TRBJ2-2_CATQNTNTGELFF	2
TRBV6-1_TRBJ2-4_CASGAGGTPVIQYF	2
TRBV3-1_TRBJ1-5_CASSHGGWQPQHF	2
TRBV5-4_TRBJ2-6_CASSLGSPGANVLTF	2
TRBV12-5_TRBJ2-5_CASGDLGGPQETQYF	2
TRBV20-1_TRBJ2-1_CSARKIAGGPGEQFF	2
TRBV2_TRBJ2-2_CASSPGPSNTGELFF	2
TRBV7-2_TRBJ2-3_CASSLIGVTLRDRGNTQYF	2
TRBV20-1_TRBJ1-1_CSAFQQGKNTEAFF	2
TRBV6-3_TRBJ1-5_CASHPPNQPQHF	2
TRBV2_TRBJ2-2_CASRPGTQNTGELFF	2
TRBV30_TRBJ2-7_CAWGKSYEQYF	2
TRBV7-2_TRBJ1-3_CASSLAWGQSSGNTIYF	2
TRBV6-3_TRBJ1-5_CASSRLPDQPQHF	2
TRBV28_TRBJ1-1_CASSFVGRRGETEAFF	2
TRBV30_TRBJ1-6_CAWRPTETYNSPLHF	2
TRBV2_TRBJ1-2_CASSAQVANYGYTF	2
TRBV14_TRBJ2-5_CASSRLTVVYQETQYF	2
TRBV3-1_TRBJ2-3_CASSQLGLASADTQYF	2
TRBV5-1_TRBJ2-3_CASSFGQITDTQYF	2
TRBV28_TRBJ1-1_CASSLWDSGTEAFF	2
TRBV20-1_TRBJ2-6_CSAPPGQGASGANVLTF	2
TRBV7-2_TRBJ2-3_CASSLGTTQYF	2
TRBV5-4_TRBJ2-7_CASSLGLAGGLYEQYF	2
TRBV6-6_TRBJ2-1_CASSRSWAGTNNEQFF	2
TRBV13_TRBJ1-1_CASSYTGNTEAFF	2
TRBV6-5_TRBJ1-6_CASSSEAGLVSPLHF	2
TRBV5-4_TRBJ2-5_CASSPGAAKETQYF	2
TRBV12-4_TRBJ2-2_CASSSHTNANTGELFF	2
TRBV10-1_TRBJ2-2_CASSEFTSGELFF	2
TRBV11-2_TRBJ2-5_CASSLVRGRGTQYF	2
TRBV19_TRBJ2-2_CASSRRTGGTGELFF	2
TRBV20-1_TRBJ2-3_CSARPTSGGQPDTQYF	2
TRBV7-3_TRBJ2-7_CASSTDRGPEYF	2
TRBV10-3_TRBJ2-3_CAIRDTTDTQYF	2
TRBV3-1_TRBJ1-2_CASSQVGYGYTF	2
TRBV19_TRBJ2-6_CASMTYSGANVLTF	2
TRBV11-2_TRBJ2-1_CASSSWAVWKFF	2

...

Total = 5909

Table S5: Top clonotype sequences and frequencies of the Treg population from all TILT patients

TRAV_TRAJ_CDR3A	#
TRAV19_TRAJ44_CALSEGWTGTASKLTF	92
TRAV2_TRAJ20_CAVNDYKLSF	83
TRAV21_TRAJ33_CAVDPSGSNYQLIW	68
TRAV41_TRAJ52_CAVLDSYGKLTF	66
TRAV12-2_TRAJ34_CAVINTDKLIF	56
TRAV3_TRAJ23_CASYNQGGKLIF	48
TRAV3_TRAJ28_CAVSLPSGAGSYQLTF	46
TRAV10_TRAJ45_CVVSGGGADGLTF	42
TRAV14DV4_TRAJ57_CAMREGPGGSEKLVF	35
TRAV19_TRAJ23_CALSEARNQGGKLIF	31
TRAV24_TRAJ27_CAFSINTNAGKSTF	30
TRAV6_TRAJ22_CAPSPSGSARQLTF	29
TRAV21_TRAJ16_CAAPSF	29
TRAV8-4_TRAJ45_CAVIPYRGGGADGLTF	27
TRAV41_TRAJ49_CAVRYTFGNQFYF	25
TRAV12-1_TRAJ17_CVVNPLKAAGNKLTF	25
TRAV12-2_TRAJ8_CAVVTGFQKLVF	21
TRAV26-2_TRAJ52_CILPLAGGTSYGKLTF	20
TRAV21_TRAJ43_CAVTDDMRF	19
TRAV14DV4_TRAJ29_CAMREGHSGNTPLVF	17
TRAV36DV7_TRAJ57_CAPRGGSEKLVF	14
TRAV4_TRAJ17_CLVGGKAAGNKLTF	13
TRAV12-3_TRAJ56_CAMSLPYDWAGANSKLTF	12
TRAV38-2DV8_TRAJ54_CAYMEIRGAQKLVF	12
TRAV22_TRAJ45_CATGGGADGLTF	12
TRAV13-1_TRAJ6_CAASRTSGGSYIPTF	11
TRAV29DV5_TRAJ42_CAASGLSIGGSQGNLIF	10
TRAV12-2_TRAJ31_CAPLDARLMF	10
TRAV36DV7_TRAJ38_CAVVPQTGNNRKLIW	10
TRAV36DV7_TRAJ17_CAPSWAAGNKLTF	10
TRAV8-3_TRAJ28_CAVAAYSGAGSYQLTF	9
TRAV21_TRAJ36_CAVEGTGANNLFF	9
TRAV24_TRAJ41_CAFVGYSGYALNF	9
TRAV24_TRAJ37_CASHGSSNTGKLIF	8
TRAV29DV5_TRAJ26_CAARYGQNFVF	8
TRAV25_TRAJ37_CAGLISSNTGKLIF	8
TRAV29DV5_TRAJ43_CAASAGNNDMRF	7
TRAV19_TRAJ27_CALSGFNTNAGKSTF	7
TRAV5_TRAJ31_CAEANARLMF	7
TRAV36DV7_TRAJ45_CAGMGADGLTF	7
TRAV13-2_TRAJ32_CAENTPWGGATNKLIF	6
TRAV12-2_TRAJ45_CAVNYYSGGGADGLTF	6
TRAV10_TRAJ40_CVVSLTSGTYKYIF	5
TRAV8-4_TRAJ40_CAVSPSSTAGTYKYIF	5
TRAV9-2_TRAJ30_CALYMNRDDKIIF	5
TRAV23DV6_TRAJ52_CAARGGTSYGKLTF	5
TRAV26-2_TRAJ21_CILRDNFNKFYF	3
TRAV35_TRAJ43_CAGHGVWNNNDMRF	3
TRAV8-4_TRAJ26_CAVTRNYGQNFVF	2
TRAV8-1_TRAJ4_CAISGGYNKLIF	2
TRAV35_TRAJ5_CAGLRGTGRRALTF	2
TRAV8-3_TRAJ37_CAVAGNTGKLIF	1
TRAV12-3_TRAJ3_CAMKESASKIIF	1
TRAV1-2_TRAJ33_CAGMDSNYQLIW	1
TRAV13-1_TRAJ15_CAASIWEGAGTALIF	1
TRAV10_TRAJ9_CVVSRAGGFKTIF	1
TRAV8-6_TRAJ3_CAVSGAASKIIF	1
TRAV21_TRAJ43_CTVTDDMRF	1
TRAV17_TRAJ22_CATRGVSGSARQLTF	1
TRAV29DV5_TRAJ40_CAARSAGRVPGTYKYIF	1
TRAV1-1_TRAJ30_CAVRDARRDDKIIF	1
TRAV13-1_TRAJ37_CAAYPGNTGKLIF	1
TRAV24_TRAJ26_CAFPYGQNFVF	1
TRAV8-6_TRAJ37_CAVSEGSSNTGKLIF	1
TRAV36DV7_TRAJ38_CAVVPQTGNIRKLIW	1
TRAV21_TRAJ30_CAVRDPLGNRDDKIIF	1
TRAV12-3_TRAJ18_CAMSDRGSTLGRLYF	1
TRAV25_TRAJ53_CAGPRGSNYKLTF	1
TRAV13-1_TRAJ27_CAASGAGKSTF	1
TRAV1-2_TRAJ33_CAVMDSNYQLIW	1
TRAV8-3_TRAJ48_CAAPNFGNEKLTF	1
TRAV30_TRAJ40_CGTALTSGTYKYIF	1
TRAV13-1_TRAJ22_CAAWYGSARQLTF	1
TRAV26-2_TRAJ28_CILLSGAGSYQLTF	1
TRAV25_TRAJ24_CAGPGGADSWGKLQF	1
TRAV2_TRAJ42_CAEGYGGSQGNLIF	1
TRAV35_TRAJ47_CAGHKYGNKLVF	1
TRAV41_TRAJ34_CAVGPSITPTSSSL	1
TRAV3_TRAJ27_CAVRDMGGTNAGKSTF	1
TRAV14DV4_TRAJ22_CAMRVVSSGSARQLTF	1
TRAV6_TRAJ48_CALHEEKLTF	1
TRAV13-2_TRAJ34_CAENKNTDKLIF	1
TRAV8-4_TRAJ54_CAVRPDQGAQKLVF	1
TRAV29DV5_TRAJ43_CAASAGNHDMRF	1
TRAV8-4_TRAJ52_CAVSAGGTSYGKLTF	1
TRAV10_TRAJ48_CVVSALSFGNEKLTF	1
TRAV12-2_TRAJ13_CAVKPPPRVTF	1
TRAV4_TRAJ4_CLVGYNKLIF	1
TRAV4_TRAJ3_CLVGDSSASKIIF	1
TRAV1-1_TRAJ23_CVRRKAYLRT	1
TRAV8-3_TRAJ22_CAVGVSGSARQLTF	1
TRAV17_TRAJ23_CATDEIMIYNQGGKLIF	1
TRAV36DV7_TRAJ38_CAVVLQTGNNRKLIW	1
TRAV3_TRAJ23_CASHNQGGKLIF	1
TRAV35_TRAJ57_CAGQNRGGSEKLVF	1
TRAV14DV4_TRAJ49_CAMRDYITGNQFYF	1
TRAV14DV4_TRAJ7_CVRGNNRLAF	1
TRAV1-2_TRAJ34_CAVRSSHLITPTSSSL	1
TRAV21_TRAJ45_CAVSWWSGGGADGLTF	1
TRAV26-1_TRAJ37_CIVRVSGLSSNTGKLIF	1
TRAV6_TRAJ8_CALDIGFQKLVF	1

Total = 2192

TRBV_TRBJ_CDR3B	#
TRBV20-1_TRBJ1-1_CSARGRAGEAFF	255
TRBV29-1_TRBJ2-7_CSVDALGDEGTYEQYF	255
TRBV28_TRBJ2-1_CATRTGPNEQFF	187
TRBV5-5_TRBJ2-1_CASSLDTTGSNEQFF	162
TRBV5-6_TRBJ1-1_CASSLGGMNTEAFF	154
TRBV5-1_TRBJ2-3_CASSYPGQRTDTQYF	151
TRBV28_TRBJ2-7_CASRARGTVPPSNEQYF	117
TRBV9_TRBJ2-1_CASSASIAGGHVYNEQFF	113
TRBV29-1_TRBJ2-7_CSVVLSDTYEQYF	110
TRBV30_TRBJ1-2_CAWRETGNNYGYTF	91
TRBV7-9_TRBJ1-1_CASSALRGEAFF	89
TRBV7-8_TRBJ1-5_CASSPDRKRNQPQHF	78
TRBV2_TRBJ2-5_CASSTPRVGQYQETQYF	69
TRBV28_TRBJ2-5_CASSLGIIQETQYF	57
TRBV4-3_TRBJ2-3_CASSQASGTSGATDTQYF	57
TRBV7-2_TRBJ1-5_CASSLGSGTHQPQHF	51
TRBV18_TRBJ1-2_CASSPYIWGWDYGYTF	50
TRBV28_TRBJ1-6_CASSLWEWSSYNSPLHF	48
TRBV28_TRBJ1-1_CASSYGQGAWAFF	48
TRBV9_TRBJ2-7_CASSEGLAGTYEQYF	46
TRBV11-2_TRBJ2-5_CASSSGGGRGGETQYF	44
TRBV7-9_TRBJ1-1_CASSLEISNTEAFF	37
TRBV7-8_TRBJ2-7_CASSLGQAYEQYF	36
TRBV5-1_TRBJ2-7_CASSFGTGGINEQYF	36
TRBV7-2_TRBJ2-7_CASSPLRAGGEQYF	35
TRBV28_TRBJ2-7_CASSFDMYSYEQYF	35
TRBV9_TRBJ2-3_CASSVLAGPTDTQYF	32
TRBV6-3_TRBJ2-2_CASRWVNTGELFF	32
TRBV13_TRBJ1-4_CASTRRAENEKLFF	30
TRBV4-2_TRBJ2-2_CASSQDGGVNTGELFF	29
TRBV7-8_TRBJ2-1_CASSLAGLYNEQFF	29
TRBV6-5_TRBJ1-2_CASSPGFGGTANYGYTF	28
TRBV19_TRBJ2-7_CASSIFGEQYF	27
TRBV28_TRBJ1-1_CASSPIGTSHLGTEAFF	27
TRBV20-1_TRBJ2-5_CSASPNQETQYF	26
TRBV6-3_TRBJ2-3_CASSKPRTYRPTDTQYF	25
TRBV7-3_TRBJ2-3_CASSPTEGATADTQYF	25
TRBV20-1_TRBJ2-7_CSARDQQGGSYEQYF	24
TRBV5-6_TRBJ1-2_CASSFEGQGPLYYGYTF	23
TRBV5-6_TRBJ1-3_CASSFRGAGNTIYF	23
TRBV6-5_TRBJ2-2_CASSLQGANTGELFF	22
TRBV5-4_TRBJ2-2_CASSFIPGTGSTGELFF	21
TRBV7-9_TRBJ1-4_CASSAPTETNEKLFF	18
TRBV6-3_TRBJ1-1_CASSYMAGTTEAFF	18
TRBV7-9_TRBJ2-1_CASNYPGQFYNEQFF	16
TRBV2_TRBJ2-7_CASGDPGDEQYF	13
TRBV28_TRBJ2-1_CASSPSHPYNEQFF	12
TRBV12-4_TRBJ2-2_CASRPWGQGGELFF	9
TRBV28_TRBJ2-7_CASSSGAGTGTAYEQYF	8
TRBV5-1_TRBJ2-5_CASSLGMEETQYF	5
TRBV11-2_TRBJ2-1_CASSLGLAGAWEQFF	5
TRBV5-5_TRBJ1-1_CASSLGGMNTEAFF	3
TRBV28_TRBJ1-1_CASGPLGNTEAFF	3
TRBV7-6_TRBJ2-7_CASSLGTGYYEQYF	2
TRBV7-9_TRBJ1-5_CASSRTAAGNQPQHF	1
TRBV28_TRBJ2-1_CASNPRTDSGIGEQFF	1
TRBV20-1_TRBJ2-7_CSAYSGPRIEQYF	1
TRBV20-1_TRBJ2-7_CSAREPARAGVEQYF	1
TRBV20-1_TRBJ2-1_CSAREGWSSYNEQFF	1
TRBV6-4_TRBJ2-3_CASSQGDLSTDTQYF	1
TRBV20-1_TRBJ2-3_CSASGSGGADTQYF	1
TRBV7-8_TRBJ2-4_CASSLGRDPAKNIQYF	1

Total = 2954

Table S6: Top clonotype sequences and frequencies of the *PRF1*$^{+}$*GZMB*$^{+}$*CD8*$^{+}$ population from all TILT patients

TRAV_TRAJ_CDR3A	#
TRAV1-2_TRAJ20_CAVRDGDYKLSF	41
TRAV1-2_TRAJ33_CAVMDSNYQLIW	37
TRAV13-1_TRAJ3_CAASMEGYSSASKIIF	29
TRAV1-2_TRAJ33_CAVRDSNYQLIW	24
TRAV1-2_TRAJ33_CAGMDSNYQLIW	23
TRAV1-1_TRAJ26_CAVYGQNFVF	19
TRAV1-2_TRAJ33_CAVPDSNYQLIW	13
TRAV1-2_TRAJ33_CAVKDSNYQLIW	12
TRAV1-2_TRAJ33_CAVTDSNYQLIW	10
TRAV1-2_TRAJ12_CAVLDSSYKLIF	6
TRAV16_TRAJ20_CAPNDYKLSF	5
TRAV1-2_TRAJ33_CAVDDSNYQLIW	4
TRAV1-2_TRAJ12_CAVKDSSYKLIF	4
TRAV5_TRAJ4_CAETGGSGGYNKLIF	4
TRAV1-2_TRAJ26_CAVYGQNFVF	2
TRAV2_TRAJ10_CAILTGGGNKLTF	2
TRAV14DV4_TRAJ23_CVWGPGRKAYLRT	2
TRAV35_TRAJ5_CAGLRGTGRRALTF	1
TRAV14DV4_TRAJ7_CAMREGPLWEQQTRF	1
TRAV12-1_TRAJ49_CVVNRNNQFYF	1
TRAV26-1_TRAJ39_CIVRAHNNAGNMLTF	1
TRAV9-2_TRAJ22_CAPEPRRQLTF	1
TRAV1-1_TRAJ20_CAVRDGDYKLSF	1
TRAV1-2_TRAJ17_CAVESGAAGNKLTF	1
TRAV1-2_TRAJ20_CAVREGDYKLSF	1
TRAV1-1_TRAJ33_CAVRDSNYQLIW	1
TRAV2_TRAJ24_CAVEDTDSWGKFQF	1
TRAV14DV4_TRAJ6_CAMRASGGSYIPTF	1
TRAV1-2_TRAJ33_CACLDSNYQLIW	1
TRAV38-2DV8_TRAJ58_CAYIPRASGSRLTF	1
TRAV1-2_TRAJ33_CAVLDSNYQLIW	1
TRAV26-2_TRAJ20_CILRDAGSNDYKLSF	1
TRAV17_TRAJ13_CATGSGGYQKVTF	1
TRAV2_TRAJ20_CAVNDYKLSF	1
TRAV1-2_TRAJ20_CAVRDGDYKLSL	1
TRAV21_TRAJ48_CAVHNFGNEKLTF	1
TRAV1-2_TRAJ12_CAVMDSSYKLIF	1
TRAV2_TRAJ26_CAVVHNYGQNFVF	1
TRAV1-2_TRAJ33_CAVEDSNYQLIW	1
TRAV8-3_TRAJ45_CAVGAHSGGGADGLTF	1
TRAV8-3_TRAJ42_CAVSKGGSQGNLIF	1
TRAV1-2_TRAJ33_CAVGDSNYQLIW	1

Total = 524

TRBV_TRBJ_CDR3B	#
TRBV6-4_TRBJ2-3_CASSDSSTDTQYF	103
TRBV28_TRBJ2-1_CASNPRTDSGIGEQFF	95
TRBV20-1_TRBJ2-3_CSASGSGGADTQYF	68
TRBV20-1_TRBJ1-1_CSAREPDRMTEAFF	60
TRBV4-2_TRBJ2-7_CASSQEGLAEPYEQYF	47
TRBV6-4_TRBJ2-3_CASSQGDLSTDTQYF	43
TRBV4-3_TRBJ2-2_CASSQGELETGELFF	39
TRBV6-3_TRBJ2-2_CASSHGQGHTGELFF	36
TRBV20-1_TRBJ2-1_CSARLADNEQFF	33
TRBV4-2_TRBJ2-5_CASSQERGGQETQYF	32
TRBV6-4_TRBJ2-1_CASSDSTSGSNEQFF	31
TRBV6-4_TRBJ2-2_CASSDRDTGELFF	30
TRBV6-4_TRBJ2-3_CASSDGASDTQYF	30
TRBV19_TRBJ2-3_CASSMTPGQGSDTQYF	30
TRBV14_TRBJ2-7_CASSQGRGLRSYEQYF	28
TRBV6-4_TRBJ2-1_CASSDSGLAGYNEQFF	28
TRBV20-1_TRBJ2-2_CSARDLSGRELFF	27
TRBV6-3_TRBJ2-1_CASSYSDYNEQFF	20
TRBV28_TRBJ1-1_CASSYGQGAWAFF	2
TRBV2_TRBJ2-7_CASGDPGDEQYF	2
TRBV6-3_TRBJ2-1_CASSRADYNEQFF	2
TRBV20-1_TRBJ1-1_CSARGRAGEAFF	2
TRBV4-3_TRBJ2-5_CASSQERGGQETQYF	2
TRBV5-4_TRBJ2-2_CASSFIPGTGSTGELFF	2
TRBV29-1_TRBJ2-7_CSVDALGDEGTYEQYF	2
TRBV20-1_TRBJ2-1_CSAREGWSSYNEQFF	1
TRBV5-5_TRBJ2-1_CASSLDTTGSNEQFF	1
TRBV7-8_TRBJ2-4_CASSLGRDPAKNIQYF	1
TRBV28_TRBJ2-7_CASSSGAGTGTAYEQYF	1
TRBV11-2_TRBJ2-1_CASSLGLAGAWEQFF	1
TRBV5-6_TRBJ2-1_CASSLDTTGSNEQFF	1
TRBV28_TRBJ2-7_CASSFDMYSYEQYF	1
TRBV30_TRBJ1-2_CAWRETGNNYGYTF	1
TRBV6-3_TRBJ2-2_CASSDRDTGELFF	1
TRBV20-1_TRBJ2-7_CSARDQQGGSYEQYF	1
TRBV29-1_TRBJ2-7_CSVEDDLSYEQYF	1
TRBV9_TRBJ2-3_CASSVLAGPTDTQYF	1
TRBV20-1_TRBJ2-7_CSAREPARAGVEQYF	1
TRBV5-4_TRBJ2-2_CASSRQTGTIDYTGELFF	1
TRBV5-1_TRBJ2-1_CASSPTRSYNEQFF	1

Total = 1618

Table S7. Top clonotype sequences and frequencies of the MAIT population from all TILT patients.
